# A CNN Feature Extraction and BKA-Optimized LSSVM Classification Method for Small-Sample Rolling Bearing Fault Diagnosis

**DOI:** 10.3390/s26165148

**Published:** 2026-08-14

**Authors:** Shiyan Sun, Yujun Shi, Quan Li, Jiwei Wang, Haifeng Lu

**Affiliations:** 1 School of Electrical Engineering, Xinjiang University, Urumqi 830017, China; 15938588246@163.com (S.S.); liquan@xju.edu.cn (Q.L.); jiweiwang@xju.edu.cn (J.W.); luhaifeng@mail.tsinghua.edu.cn (H.L.); 2 Xinjiang E&T Research Center for Operation and Control of New Power System with High Penetration of Wind and Solar Energy, Xinjiang University, Urumqi 830017, China

**Keywords:** black-winged kite algorithm, continuous wavelet transform, least squares support vector machine, small-sample diagnosis, bearing fault diagnosis, convolutional neural network

## Abstract

Rolling bearings are indispensable elements in mechanical equipment, and their condition is closely related to system reliability and operational safety. To enhance diagnostic performance with limited samples and reduce the dependence on manual parameter selection, this study proposes a fault diagnosis approach that combines Continuous Wavelet Transform (CWT), Convolutional Neural Network (CNN), Black-winged Kite Algorithm (BKA), and Least Squares Support Vector Machine (LSSVM). The original 1-D vibration signals are first processed by CWT to obtain 2-D time–frequency representations. CNN is then employed to learn deep fault-sensitive features, which are subsequently fed into LSSVM for state classification. To further improve classification performance, BKA is used to automatically search for the optimal LSSVM parameters, with validation accuracy adopted as the fitness criterion. Experiments conducted on three public bearing datasets, namely CWRU, JNU, and SEU, indicate that the proposed method outperforms CNN, CNN-SVM, and CNN-BiGRU under small-sample conditions. In addition, t-SNE results show more distinct feature clusters, while BKA exhibits faster convergence and better global search capability than Particle Swarm Optimization (PSO) and Genetic Algorithm (GA).

## 1. Introduction

Rolling bearings constitute indispensable transmission components in modern mechanical systems, directly affecting the overall operating performance of the machinery, operational reliability, and long-term service stability. They execute essential mechanical functions, including supporting dynamic and static loads, maintaining precise component positioning, transmitting torque and forces, and minimizing frictional losses between moving parts. The operational condition of rolling bearings directly correlates with equipment efficiency, functional integrity, and lifecycle durability. Hence, the implementation of rapid and reliable fault diagnosis for bearings is of paramount importance—not only for ensuring safe and efficient machinery operation but also for substantially reducing unplanned maintenance expenditures, preventing catastrophic system failures, and optimizing overall production throughput. Over prolonged service periods, bearings are prone to multiple forms of deterioration such as material fatigue, abrasive wear, surface pitting, lubrication breakdown, and contamination ingress, which collectively contribute to performance deterioration and eventual functional failure. Under such fault conditions, the vibration signals generated by bearings encapsulate critical information pertaining to fault type, severity, and location, thereby serving as direct and informative indicators of bearing health. Through signal processing of these vibration signatures, discriminative fault features can be effectively extracted, facilitating the early diagnosis of incipient faults and the accurate identification of developed faults in rolling bearings. This diagnostic process is fundamental to enabling proactive maintenance strategies, rapid fault identification, efficient troubleshooting, minimization of operational downtime, and preservation of mechanical system integrity in industrial applications.

For bearing fault diagnosis, vibration signal analysis has been widely adopted as a mainstream technical approach. Conventional time–frequency analysis techniques, including Fast Fourier Transform (FFT) [1,2], Gramian Angular Field (GAF) [3,4], and Variational Mode Decomposition (VMD) [5,6], can reveal the time–frequency structure of signals to a certain extent. When applied to non-stationary and nonlinear signals, these methods are often restricted by fixed time–frequency resolution and exhibit insufficient capability in capturing transient impact features. To address this, this paper introduces CWT [7,8] as the signal processing tool. This method, with its adjustable time–frequency window, can better adapt to the analysis requirements of non-stationary signals and is particularly suitable for extracting transient impact features common in bearing faults.

With the rapid development of AI, deep learning diagnosis methods have demonstrated considerable promise. Because of the ability to learn fault-related representations from raw or transformed signals automatically, CNNs have been widely studied in bearing fault diagnosis [9,10,11,12]. Although CNNs can learn hierarchical and discriminative representations from raw data automatically, the softmax classifier commonly used at the output layer may not provide sufficient generalization ability for complex and highly nonlinear classification tasks, especially when labeled samples are limited. This is mainly because softmax operates on high-dimensional feature vectors and may easily overfit the training data under small-sample conditions, which weakens its performance on unseen or noise-contaminated samples. To address this problem and improve classification accuracy and robustness, many studies separate feature learning from the final classification stage. In such a hybrid strategy, a CNN serves as a nonlinear feature extraction module to generate compact and informative feature representations, which are then input into traditional machine learning classifiers, such as k-Nearest Neighbors, Random Forests, or Support Vector Machines (SVM). These classifiers often show better generalization performance in limited-sample scenarios.

The effectiveness of hybrid diagnostic models has been verified in several studies. Niu et al. [13] developed an improved discriminative feature-learning deep residual CNN by incorporating domain knowledge and attention mechanisms. To improve the extraction of fault-sensitive features, channel attention and non-local attention mechanisms were incorporated, while dual classifiers were adopted for multi-task diagnosis, thereby improving bearing fault type recognition and fault location identification. Wang et al. [14] proposed a diagnosis method based on symmetrical dot pattern visualization and a squeeze-and-excitation CNN. By optimizing the parameters for SDP image generation and designing an SE-CNN model to adaptively assign weights to feature channels, the method strengthened the representation of key fault information and achieved accurate bearing state diagnosis. Li et al. [15] presented a few-shot fault diagnosis approach that integrates CWT-based time–frequency imaging, KAN-CNN feature extraction, and diffusion-model-based sample augmentation, which improved diagnostic accuracy and robustness with limited samples. Han et al. [16] designed a bearing fault diagnosis approach combining CNN and Transformer. Through redundancy filtering mechanisms and spatial-channel reconstruction, the network adaptively selected useful features in different task branches and realized simultaneous identification of fault type and fault severity under noisy conditions.

Recent studies have further explored hybrid learning and automated optimization for intelligent condition monitoring. CNN–LSTM and CNN–BiLSTM frameworks have been employed to combine local feature extraction with temporal dependency modeling, while data augmentation and pretrained CNNs have been investigated to improve feature learning under limited-data conditions. In addition, Bayesian optimization and hybrid metaheuristic algorithms have been introduced to automate hyperparameter selection and balance global exploration and local exploitation [17,18,19,20,21]. Cheng et al. employed knowledge distillation and adaptive feature selection to improve diagnostic performance and model compactness under imbalanced samples, whereas Zhao et al. developed a variational gated hierarchical memory network to enhance feature learning and diagnostic robustness in high-noise environments [22,23].

Although these methods have improved diagnostic performance to some extent, practical problems remain, including limited generalization ability, noise sensitivity, and unstable performance when training samples are insufficient. Against this background, SVM has received considerable attention in fault diagnosis because of its statistical learning foundation and strong small-sample classification ability [24]. As a representative extension of SVM, LSSVM reformulates the original optimization problem by converting inequality constraints into equality constraints and takes the squared error term as the objective loss. In this way, the complex quadratic programming problem is reduced to the solution of linear equations, which reduces computational complexity and improves training efficiency. While maintaining the generalization advantage of SVM, LSSVM is more convenient for practical implementation and system integration. Therefore, it is suitable for combining with CNN to classify deep features extracted by CNN in an efficient and robust manner. In recent years, LSSVM has shown promising application prospects in various fault-diagnosis tasks [25,26,27].

Nevertheless, the effectiveness of LSSVM is largely influenced by two essential hyperparameters: the regularization parameter γ and the width parameter $\sigma^{2}$ of the Radial Basis Function (RBF) kernel. Here, γ is used to balance model complexity and training error, while $\sigma^{2}$ governs the distribution characteristics of samples in the feature space. Inappropriate parameter selection can lead to underfitting or overfitting, adversely affecting diagnostic accuracy. Common parameter determination methods mainly include grid search, random search, and experience-based trial-and-error. These methods still have certain limitations in practical application: although systematic and comprehensive, grid search incurs significantly increased computational costs as parameter dimensions rise, often resulting in low efficiency. Random search improves search efficiency to some extent, but its lack of directional exploration may struggle to guarantee optimization stability. Methods relying on manual trial-and-error are more subjective and typically cannot ensure finding a global optimum.

To automate and intelligently optimize LSSVM parameters, meta-heuristic optimization algorithms are widely adopted. Among them, PSO [28,29] and GA [30,31] are two classic representatives. However, when solving high-dimensional, nonlinear, multimodal function optimization problems, PSO and GA may exhibit limitations such as premature convergence or inefficient search.

Recently, several novel metaheuristic algorithms have been developed to overcome the aforementioned limitations. The BKA was proposed in 2024 [32]. It mimics the distinctive behaviors of the black-winged kite, including diving attacks and seasonal migration. BKA achieves extensive global exploration through its attack behavior, carries out refined local exploitation via migration behavior integrated with a dynamic leadership mechanism, and incorporates Cauchy mutation to enhance population diversity. This distinctive search mechanism helps achieve a balanced compromise between convergence speed and global optimization capability, offering a new solution for complex optimization problems.

Unlike conventional CNN–classifier frameworks in which classifier parameters are manually specified or determined by grid search, the proposed method employs BKA to adaptively optimize the regularization parameter and RBF-kernel width of LSSVM. BKA is derivative-free and is therefore suitable for searching the nonlinear validation-performance surface of LSSVM. Moreover, its attack and migration mechanisms, dynamic leadership strategy, and Cauchy mutation help maintain population diversity and balance global exploration with local exploitation, thereby reducing the risk of premature convergence. Since BKA optimizes only two LSSVM parameters after CNN feature extraction, the optimization space remains compact without increasing the complexity of CNN training.

By leveraging the powerful feature representation capability of CNN and the strong generalization performance of LSSVM, especially their suitability for small-sample scenarios, this paper introduces an integrated bearing fault diagnosis method, namely CWT-CNN-BKA-LSSVM. This method is designed to enhance diagnostic accuracy under data-limited conditions while systematically addressing the inherent limitations of empirical parameter tuning in LSSVM, which often compromises optimization efficiency and stability. By implementing an intelligent parameter optimization mechanism, the proposed method enhances classification accuracy while enhancing its applicability to practical scenarios.

The remainder of this paper is structured as follows. Section 2 focuses on the methodological foundation of the proposed CWT-CNN-BKA-LSSVM framework. It clarifies how time–frequency representation, deep feature extraction, optimized LSSVM classification, and BKA-based parameter optimization are integrated to improve fault diagnosis under limited-sample conditions. Section 3 validates the effectiveness of the proposed method through experiments on three public bearing datasets. Emphasis is placed on its diagnostic accuracy, small-sample generalization ability, feature separability, and optimization efficiency in comparison with representative benchmark models and optimization algorithms. Section 4 summarizes the main findings and discusses the potential value of the proposed method for intelligent bearing fault diagnosis under small-sample conditions.

The main contributions of this study are outlined as follows:

(1) A bearing fault diagnosis method named CWT-CNN-BKA-LSSVM is developed. In this method, CNN is responsible for learning deep and discriminative fault features, whereas LSSVM performs the final classification task. By combining the feature representation ability of deep neural networks with the efficient classification capability of LSSVM, this method improves generalization performance when only limited training samples are available.
(2) BKA is introduced to adaptively tune the key parameters of LSSVM. Compared with traditional parameter selection methods, which often rely on manual experience, require considerable tuning time, and may fall into local optima, the proposed optimization scheme enables more effective parameter searching and improves both the diagnostic performance and convergence efficiency of the model.
(3) The effectiveness of the proposed method is verified on multiple public bearing datasets and benchmarked against representative fault diagnosis approaches. Superior diagnostic accuracy, better small-sample adaptability, and clearer feature separation are observed in the proposed method, confirming its potential for reliable industrial fault diagnosis.

## 2. Introduction of the Proposed Method

### 2.1. Framework of CWT-CNN-BKA-LSSVM

**Figure 1:**
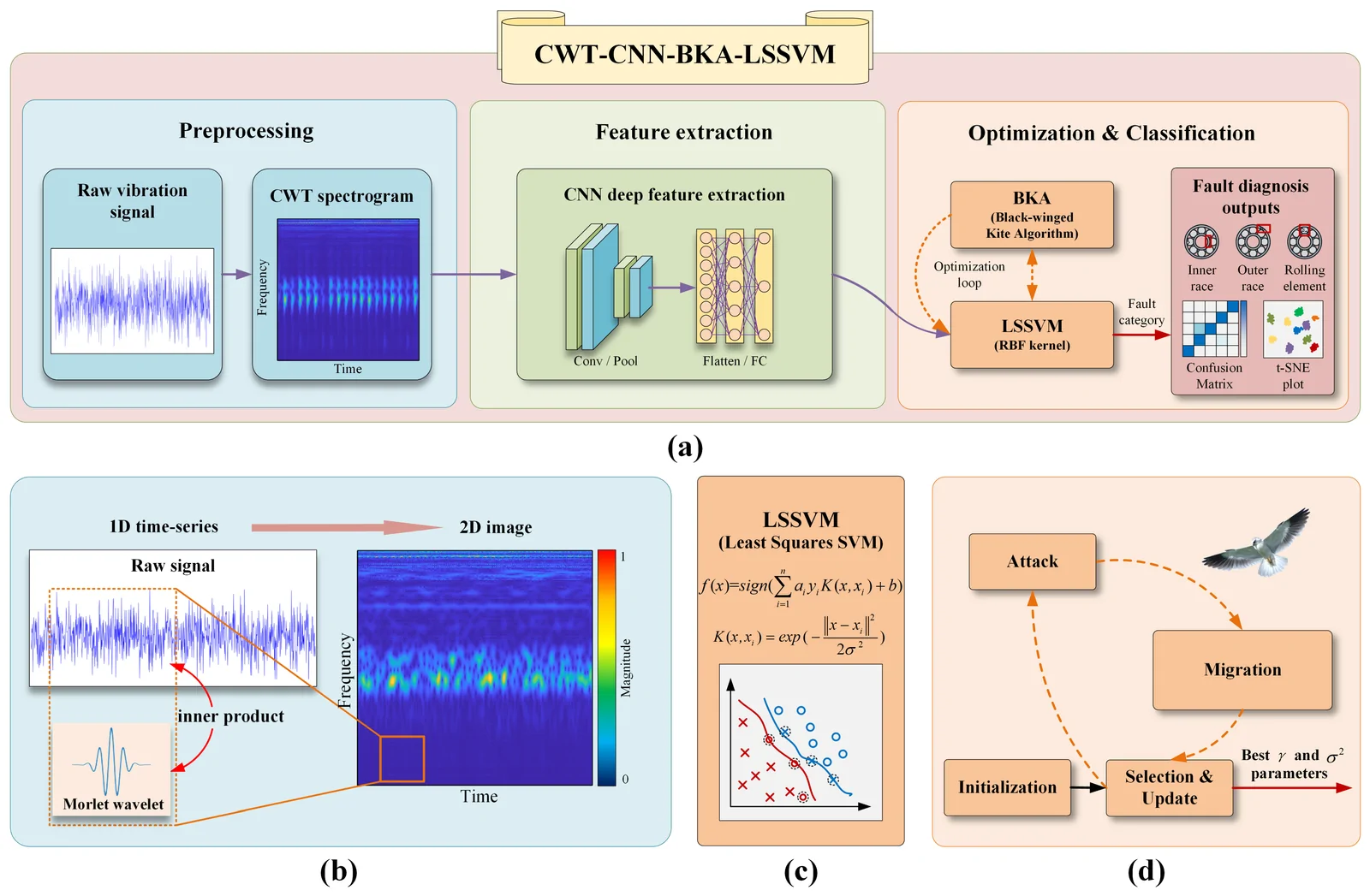
Schematic diagram of CWT-CNN-BKA-LSSVM. (**a**) Overall framework. (**b**) CWT. (**c**) LSSVM classification. (**d**) BKA optimization.

As illustrated in Figure 1a, the proposed method is generally composed of three main stages: data preprocessing, feature extraction, parameter optimization & diagnostic result generation.

As shown in Figure 1b, CWT is applied in the preprocessing stage to map the original 1-D bearing vibration signals into 2-D time–frequency representations. This transformation enables fault-related characteristics to be described from both temporal and spectral perspectives, which enables the subsequent deep feature learning process to access more comprehensive information. In the following feature-extraction stage, shown in Figure 1a, a CNN is constructed and trained to automatically capture high-level fault representations from the generated time–frequency images. The learned features are then delivered to an LSSVM classifier for fault state recognition, as presented in Figure 1c. To enhance the classification capability of LSSVM, BKA is further incorporated, as shown in Figure 1d, to adaptively optimize γ and $\sigma^{2}$, where the validation-set accuracy is used as the fitness value. This optimization strategy helps overcome the low efficiency and local optimum issues commonly encountered in conventional parameter selection methods. Finally, during the diagnosis output stage, the optimized CNN-LSSVM model is applied to the test samples to identify their corresponding fault types, thus completing the diagnostic procedure from signal input to fault identification.

### 2.2. Continuous Wavelet Transform

CWT employs an adaptive time–frequency window. By avoiding the fixed-window constraint of short-time Fourier transform, it is better suited to analyzing transient impact signals associated with bearing faults.

A wavelet is derived from a mother wavelet ψ(t) through translation and scaling, as shown in Equation (1):

(1) $$\psi_{a,b}\left(t\right)=\frac{1}{\sqrt{a}}\psi \left(\frac{t-b}{a}\right)$$

where *a* denotes the scale factor and *b* represents the time-shift parameter. Given a finite-energy signal x(t), its CWT associated with the mother wavelet ψ(t) can be expressed as a Hilbert-space inner product:

(2) $${\mathrm{CWT}}_{x}\left(a,b\right)=\frac{1}{\sqrt{a}}\int x\left(t\right)\psi^{*}\left(\frac{t-b}{a}\right)dt=\int x\left(t\right)\psi_{a,b}^{*}\left(t\right)dt=\langle x\left(t\right),\psi_{a,b}\left(t\right)\rangle$$

To effectively reveal the characteristic components of vibration signals, an appropriate mother wavelet must be selected. Although there is no standard method for choosing a mother wavelet for different tasks at present, a suitable wavelet can be selected based on the actual signal characteristics. Rolling bearing fault signals exhibit significant periodic transient impulses. The Morlet wavelet function closely resembles the shape of impulse signals, and the cmor wavelet—the complex form of the Morlet wavelet—possesses better adaptive capability. Therefore, this paper selects the cmor3-3 wavelet as the mother wavelet function for CWT. Its definition is given by Equation (3):

(3) $$\psi \left(t\right)=\frac{1}{\sqrt{\pi f_{b}}}e^{2i\pi f_{c}t}e^{-\frac{t^{2}}{f_{b}}}$$

where $f_{c}$ represents the wavelet center frequency, which specifies the oscillatory behavior of the wavelet at the reference scale. By tuning $f_{c}$, the wavelet can be adjusted to match the frequency range of interest. The parameter $f_{b}$ denotes the bandwidth factor and balances the time and frequency resolutions. A larger $f_{b}$ extends the wavelet in the time domain and compresses its spectrum, leading to higher frequency resolution, whereas a smaller $f_{b}$ results in the reverse behavior.

### 2.3. Black-Winged Kite Algorithm

According to the original formulation, BKA consists of three main stages: population initialization, attacking behavior, and migration behavior. Hovering observation describes the biological behavior of black-winged kites before attacking prey and is incorporated into the attacking mechanism rather than being treated as an independent position-update operator. The attacking operator is mainly responsible for search-space exploration, whereas the migration operator combines a dynamic leader strategy and Cauchy mutation to guide the population toward promising regions and maintain diversity.

(1) Initialization Stage
  In BKA, population initialization begins with the generation of multiple random candidate solutions. The positions of black-winged kites are then expressed in matrix form as follows:
  (4) $$BK=\left[\begin{array}{cccc} BK_{1,1} & BK_{1,2} & \cdots & BK_{1,\dim}\\ BK_{2,1} & BK_{2,2} & \cdots & BK_{2,\dim}\\ \vdots & \vdots & \ddots & \vdots \\ BK_{pop,1} & BK_{pop,2} & \cdots & BK_{pop,\dim} \end{array}\right]$$
  where pop represents the population size, dim denotes the dimensionality of the optimization problem, and $BK_{i,j}$ is the position component of the *i*-th black-winged kite in the *j*-th dimension.
  Subsequently, the position of each black-winged kite is initialized based on a uniform distribution:
  (5) $$X_{i}=BK_{lb}+rand\left(BK_{ub}-BK_{lb}\right)$$
  where i∈{1,2,…,pop}, $BK_{lb}$ and $BK_{ub}$ are the lower and upper limits of the *i*-th black-winged kite along dimension *j*, respectively, and rand is a uniformly distributed random value in [0,1].
  At the initialization stage, BKA identifies the best-performing individual in the initial population and assigns it as the leader $X_{L}$, representing the optimal position.
(2) Attack Behavior
  The attack behavior of BKA is mainly used to expand the search range and enhance its global exploration capability. Its mathematical model is given as follows:
  (6) $$y_{t+1}^{i,j}=\left\{\begin{array}{cc} y_{t}^{i,j}+n\left(1+\sin(r)\right)\times y_{t}^{i,j}, & p<r,\\ y_{t}^{i,j}+n\times \left(2r-1\right)\times y_{t}^{i,j}, & \mathrm{else}. \end{array}\right.$$
  (7) $$n=0.05\times e^{-2\times {\left(\frac{t}{T}\right)}^{2}}$$
  where $y^{i,j}$ denotes the coordinate of the *i*-th black-winged kite in dimension *j*, *r* is a random value within [0,1], *p* is a constant equal to 0.9, *T* represents the maximum number of iterations, and *t* indicates the current iteration index.
(3) Migration Behavior
  In this phase, BKA adjusts the positions of individuals by using a dynamic leader-selection strategy. If the current population has poorer fitness than a randomly selected population, the leader is replaced and incorporated into the migration group. In contrast, when the current population achieves better fitness, the original leader continues to guide the search direction. This adaptive leadership mechanism enables the algorithm to select superior leaders dynamically, thereby improving its global exploration capability. The migration behavior of the black-winged kite is mathematically described as follows:
  (8) $$y_{t+1}^{i,j}=\left\{\begin{array}{cc} y_{t}^{i,j}+C\left(0,1\right)\times \left(y_{t}^{i,j}-L_{t}^{j}\right), & F_{i}<F_{ri}\\ y_{t}^{i,j}+C\left(0,1\right)\times \left(L_{t}^{j}-m\times y_{t}^{i,j}\right), & \mathrm{else} \end{array}\right..$$
  (9) $$m=2\times \sin\left(r+\frac{\pi }{2}\right)$$
  where $L_{t}^{j}$ denotes the leader score in the *j*-th dimension at iteration *t*. $y_{t}^{i,j}$ and $y_{t+1}^{i,j}$ are the position components of the *i*-th black-winged kite in dimension *j* before and after updating, respectively. $F_{i}$ is the fitness value of the *i*th individual, and $F_{r}i$ is the fitness value of a randomly selected individual. Moreover, C(0,1) refers to the Cauchy mutation operator.

### 2.4. CNN

In this study, CNN is adopted as the core module for deep feature learning. Instead of relying on handcrafted feature extraction, CNN learns representative patterns directly from the input time–frequency images through a layered architecture. The convolutional layers are used to capture local fault-sensitive patterns, the pooling layers reduce redundant information and improve feature compactness, and the fully connected layers integrate the learned representations to support subsequent classification. The convolution operation is defined as follows:

(10) $$x_{j}^{l}=\sigma \left(\sum_{i\in M_{j}}x_{i}^{l-1}\times w_{ij}^{l}+b_{j}^{l}\right)$$

where $x_{j}^{l}$ denotes the feature map generated by the *j*-th convolution kernel at layer *l*, and its input is derived from the output of the previous layer (l−1). σ represents the nonlinear activation function, $M_{j}$ indicates the set of input feature maps connected to the *j*-th kernel, $w_{ij}^{l}$ is the convolution weight between the *i*-th input feature map and the *j*-th output feature map in layer *l*, and $b_{j}^{l}$ denotes the bias corresponding to the *j*-th kernel.

**Figure 2:**
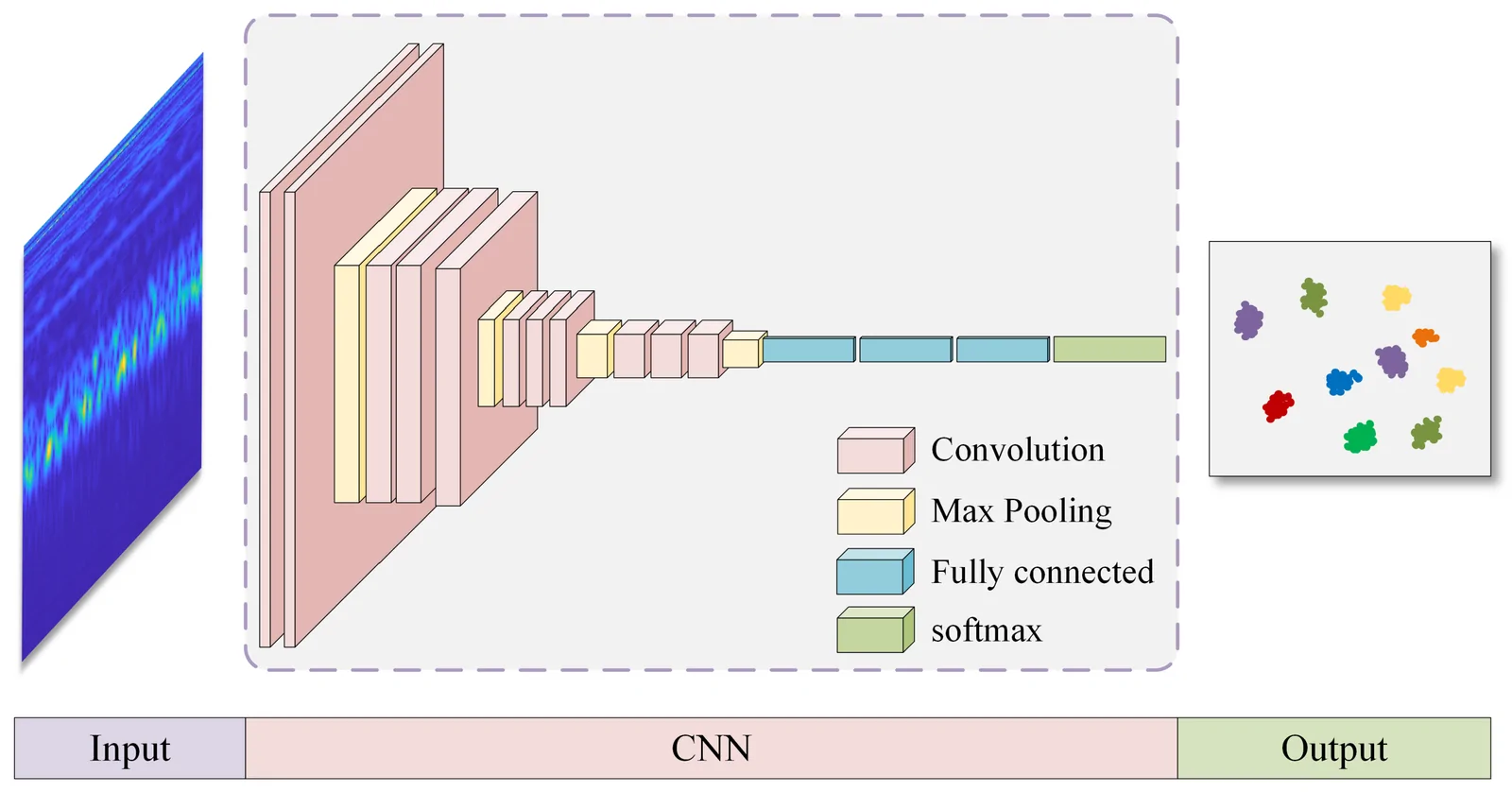
Basic structure of CNN.

The pooling layer is used to downsample feature maps through operations such as maximum pooling or average pooling. This process reduces feature dimensionality and computational burden while retaining key information for subsequent feature learning. Subsequently, the features generated by the pooling layer are integrated through the fully connected layer and then delivered to the softmax classifier for final category prediction. The typical CNN structure is illustrated in Figure 2.

### 2.5. LSSVM

LSSVM aims to identify an optimal regression function by minimizing the squared error criterion, as formulated in Equation (11):

(11) $$\min\sum_{i=1}^{n}e_{i}^{2}=\min\sum_{i=1}^{n}{\left(Y_{i}-{\hat{Y}}_{i}\right)}^{2}$$

where $e_{i}$ is the error term, $Y_{i}$ represents the actual value, and ${\hat{Y}}_{i}$ denotes the predicted value.

Within the LSSVM formulation, the error variables are introduced into the dual optimization problem, while the original inequality constraints are replaced by equality constraints. Meanwhile, the squared error term is embedded in the objective function, and the final solution is obtained according to the Karush–Kuhn–Tucker (KKT) conditions. This approach effectively reduces computational demands while simplifying the complexity of problem solving. The optimization function of LSSVM is given by Equation (12):

(12) $$\min_{\omega ,b,e}J\left(\omega ,b,e\right)=\frac{1}{2}{∥\omega ∥}^{2}+\frac{\gamma }{2}\sum_{i=1}^{n}e_{i}^{2},$$

(13) $$s.t.\;y_{i}\left[\omega^{T}\phi \left(x_{i}\right)+b\right]=1-e_{i},\;i=1,2,\ldots ,n,$$

where $x_{i}$ is the input feature vector, $y_{i}\in \left\{-1,+1\right\}$ is the class label, ω is the weight vector in the feature space, *b* is the bias term, and γ is the regularization parameter.

The Lagrangian function is introduced as follows:

(14) $$L\left(\omega ,b,e,\alpha \right)=\frac{1}{2}{∥\omega ∥}^{2}+\frac{\gamma }{2}\sum_{i=1}^{n}e_{i}^{2}-\sum_{i=1}^{n}\alpha_{i}\left\{y_{i}\left[\omega^{T}\phi \left(x_{i}\right)+b\right]-1+e_{i}\right\},$$

where *b* denotes the bias term, γ represents the regularization parameter, α is the Lagrange multiplier, and $\alpha_{i}$ indicates the multiplier corresponding to the *i*-th sample.

By setting the partial derivatives with respect to ω, *b*, α, and *e* to zero, the model parameters can be determined through the solution of the resulting linear equation system. Accordingly, the final classification decision function is given as

(15) $$f\left(x\right)=\mathrm{sign}\left[\sum_{i=1}^{n}\alpha_{i}y_{i}K\left(x,x_{i}\right)+b\right].$$

where $K(x,x_{i})$ denotes the kernel function, which projects the input samples from the original low-dimensional space into a high-dimensional feature space. Commonly used kernel functions include polynomial, radial basis function (RBF), and Sigmoid kernels. Among them, the RBF kernel is widely adopted because of its strong nonlinear mapping ability. Therefore, the proposed bearing fault diagnosis model selects the RBF kernel for LSSVM classification, and its mathematical expression is defined in Equation (16):

(16) $$K\left(x,x_{i}\right)=\exp\left(-\frac{{∥x-x_{i}∥}^{2}}{2\sigma^{2}}\right)$$

where $\sigma^{2}$ is the RBF kernel parameter, which controls the width of the kernel function.

### 2.6. CNN-LSSVM

**Figure 3:**
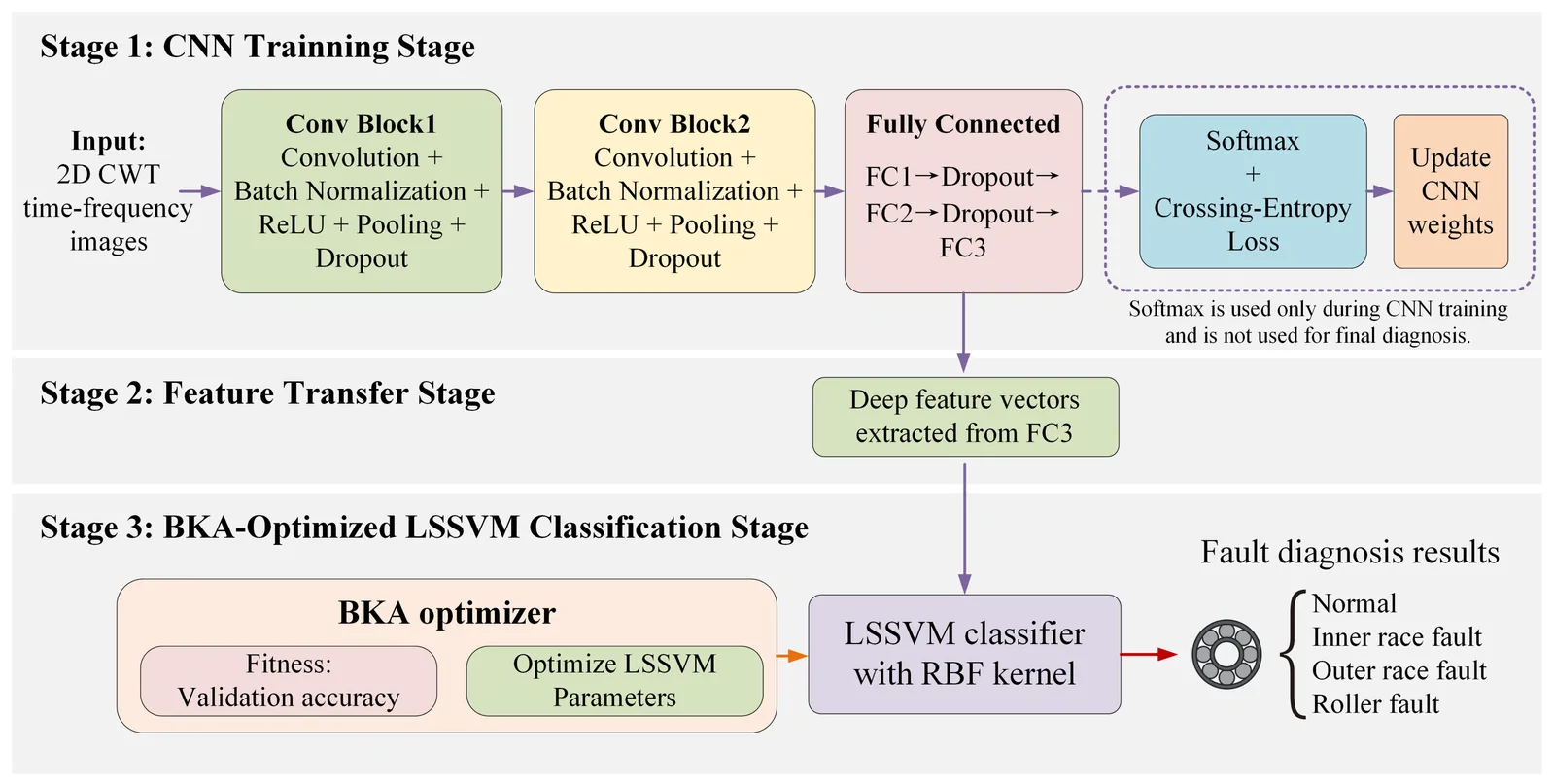
CNN-LSSVM architecture.

As shown in Figure 1a, the proposed method adopts a cascaded framework that combines deep feature learning with an intelligent classification module. In this framework, the vibration signals processed by continuous wavelet transform are first input into a CNN for deep feature extraction. The obtained high-level feature representations are then transferred to an LSSVM for accurate fault recognition, as shown in Figure 3.

The CNN module is designed with a stacked structure, including an input layer, two convolution–batch-normalization–ReLU–pooling blocks, and three FC layers. The first and second convolutional blocks contain 16 kernels of size 3×3 and 32 kernels of size 5×5, respectively, enabling the network to learn fault features from low-level to high-level representations. Pooling layers are introduced to reduce feature dimensionality. After feature flattening, the resulting vector is passed through three FC layers for feature fusion, while the output dimension of the final layer is configured according to the total number of fault classes.

During CNN training, a softmax layer and a classification layer are added after the FC layers, while the network parameters are learned through the minimization of the cross-entropy loss function. This allows the CNN to obtain an initial classification ability. However, in the feature transfer stage, the probability output generated by softmax is not used. Instead, the activation values of the last fully connected layer before softmax, namely Full Connection Layer 3, are extracted as the original deep feature representation. This arrangement is adopted for two reasons. On the one hand, softmax maps logits into a normalized probability distribution, which may weaken part of the discriminative information among different classes. On the other hand, LSSVM with an RBF kernel can model the distribution of the original feature space more effectively. Therefore, CNN is employed primarily for nonlinear feature extraction instead of directly performing the final classification.

The high-level features extracted by CNN are subsequently input into the LSSVM classifier to complete fault identification. The LSSVM’s performance is largely determined by γ and $\sigma^{2}$. Inappropriate parameter settings may cause underfitting or overfitting, thus reducing diagnostic accuracy and generalization performance. To overcome the inefficiency and local optimum problems of empirical tuning and grid search, the BKA is introduced to adaptively optimize γ and $\sigma^{2}$. As a result, the CNN-LSSVM model establishes a complete diagnostic pipeline of “signal input–deep feature extraction–feature transfer–optimized classification”. This architecture integrates CNN’s strong feature representation ability with the superior generalization performance of LSSVM under small-sample conditions, thereby enabling accurate identification of multiple bearing fault conditions.

### 2.7. LSSVM Parameter Optimization Based on the BKA Algorithm

**Figure 4:**
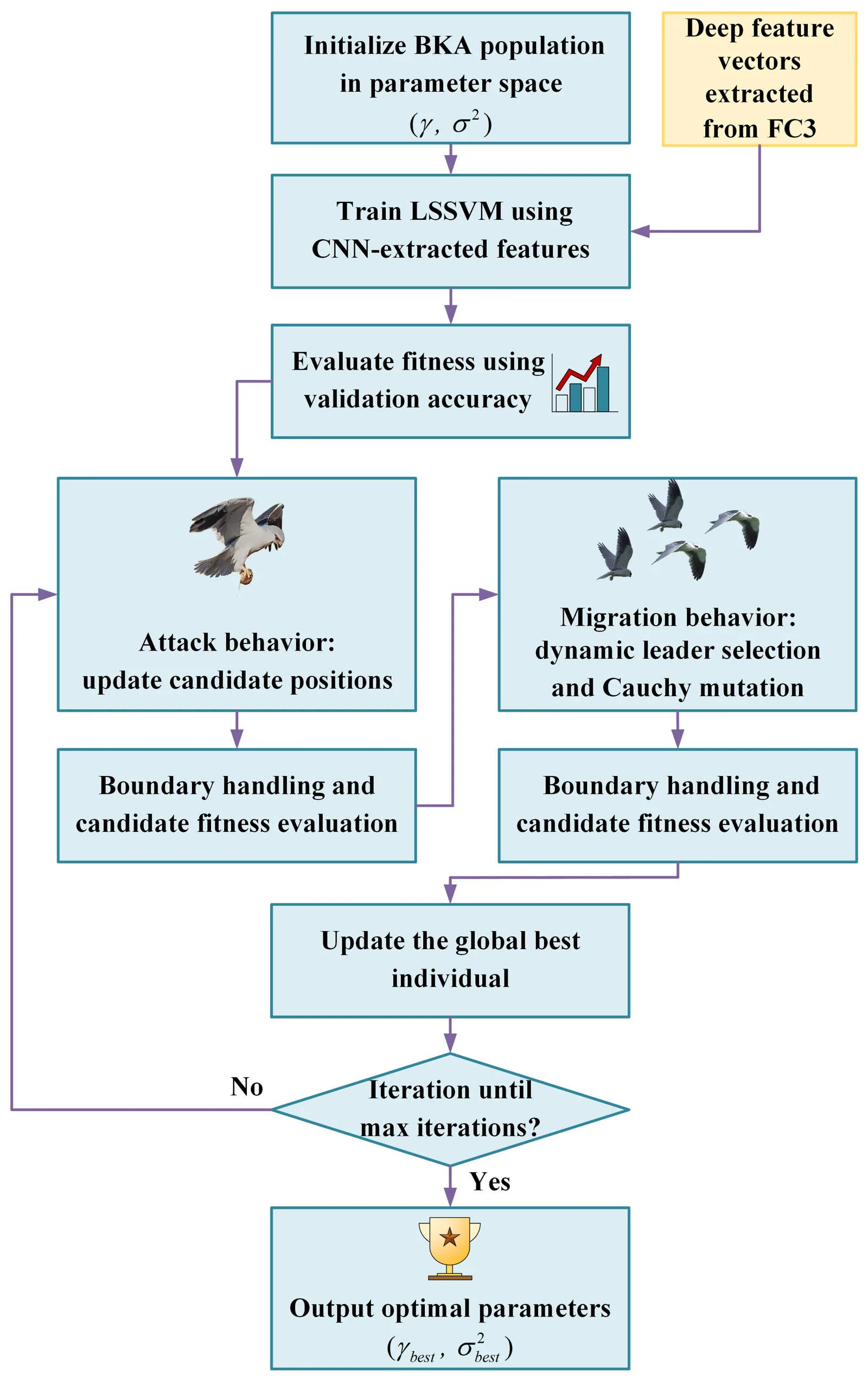
BKA optimization flowchart.

As shown in Figure 1a, BKA is employed in this study to optimize the LSSVM’s parameters. The fitness function is the classification accuracy on the validation set to evaluate the model performance under different parameter combinations. For a given parameter combination $\left(\gamma ,\sigma^{2}\right)$, a higher validation accuracy of LSSVM indicates a better parameter combination. Therefore, the optimization objective of BKA is to search within the predefined range for the parameter combination that yields the highest validation accuracy. The optimization flowchart is shown in Figure 4.

The optimization steps are as follows:

(1) Initialization
  The BKA population size, maximum iterations, and search boundaries for γ and $\sigma^{2}$ in LSSVM are defined. Then, a group of individuals is randomly distributed over the search space, with each individual encoding the candidate values of the two parameters.
(2) Fitness Evaluation
  For each individual (i.e., each set of $\left(\gamma ,\sigma^{2}\right)$ parameters), train an LSSVM model using these parameters and calculate the classification accuracy on the validation set. For each individual, its fitness is evaluated using the corresponding classification accuracy. The best-performing individual in the initial population is then chosen to be the leader.
(3) Execute Attack Behavior
  Update all individuals’ positions according to BKA’s attack strategy formula to perform global exploration.
(4) Execute Migration Behavior
  Update individuals’ positions based on BKA’s migration strategy formula. This stage incorporates a dynamic leadership mechanism to guide the population in exploitative search.
(5) Iteration and Termination
  Repeat steps 2 to 4. After each iteration, record the current global best fitness value and its corresponding parameters. Once the predefined maximum iteration number is reached, the optimization procedure stops and returns the best historical parameter pair $\left(\gamma ,\sigma^{2}\right)$.
(6) Model Application
  Substitute the optimal parameters $\gamma_{best}$ and $\sigma_{best}^{2}$ obtained by BKA into the LSSVM model, retrain using all training data, and finally apply the model to fault classification tasks on the test set, thereby constructing a high-performance CNN-BKA-LSSVM fault diagnosis model.

### 2.8. Training, Validation, and Testing Protocol

To ensure a fair evaluation and prevent information leakage during hyperparameter optimization, a three-stage training–validation–testing protocol was adopted. For each subset, the original vibration signals were first transformed into two-dimensional time–frequency images using CWT. The CNN feature extractor was then trained exclusively on the training set by minimizing the classification loss through backpropagation. Neither the validation set nor the test set was used to update the CNN parameters. After convergence, the CNN weights were fixed throughout the subsequent optimization and evaluation procedures.

The trained CNN was subsequently used to extract deep feature representations from the training, validation, and test sets independently. During BKA optimization, each candidate individual represented an LSSVM hyperparameter pair $\left(\gamma ,\sigma^{2}\right)$. For each candidate pair, the LSSVM was trained using only the CNN features from the training set, while its classification accuracy on the fixed validation set was used as the fitness value. Thus, the validation set was used exclusively for hyperparameter selection and was not involved in CNN training or final performance evaluation.

After the optimization process was completed, the optimal parameter pair was used to retrain the LSSVM with the training features. The resulting classifier was then evaluated once on the independent test set to obtain the final diagnostic results. The test set remained completely isolated from CNN training, BKA optimization, and parameter selection, thereby providing an unbiased assessment of the proposed CWT–CNN–BKA– LSSVM method.

## 3. Experimental Validation

### 3.1. Dataset Description

In this study, selected data from different types of bearings under various operating conditions in the Case Western Reserve University (CWRU), Jiangnan University (JNU), and Southeast University datasets (SEU) are used for experimental validation.

#### 3.1.1. Case Western Reserve University Dataset

**Figure 5:**
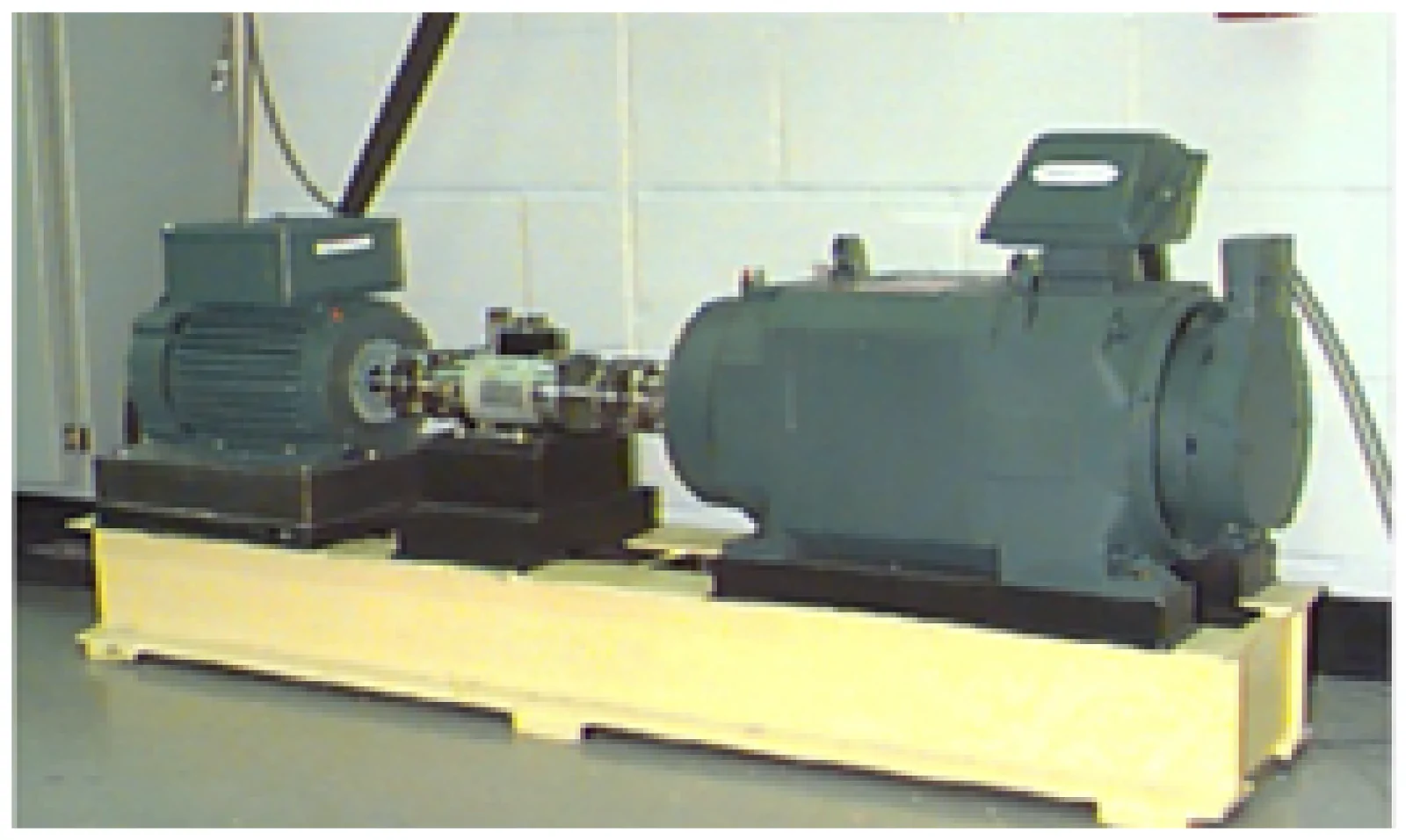
Rolling bearing test rig of CWRU [33].

The CWRU rolling bearing experimental platform [33] is shown in Figure 5. The test system is composed of, from left to right, a 1.5 kW motor, a torque transducer/encoder, and a dynamometer. Vibration acceleration signals of the tested faulty bearing are acquired by accelerometers installed on the fan-end and drive-end bearing housings. A 16-channel data acquisition system is used for vibration signal recording, while the torque transducer measures the motor power and rotational speed. The drive-end bearing adopts a 6205-2RS JEM SKF deep-groove ball bearing, whereas the fan-end bearing uses a 6203-2RS JEM SKF deep-groove ball bearing. This dataset contains vibration data collected under four different load conditions and includes 95 health states in total, consisting of eight normal states, 53 outer-race fault states, 23 inner-race fault states, and 11 rolling-element fault states.

#### 3.1.2. Jiangnan University Dataset

**Figure 6:**
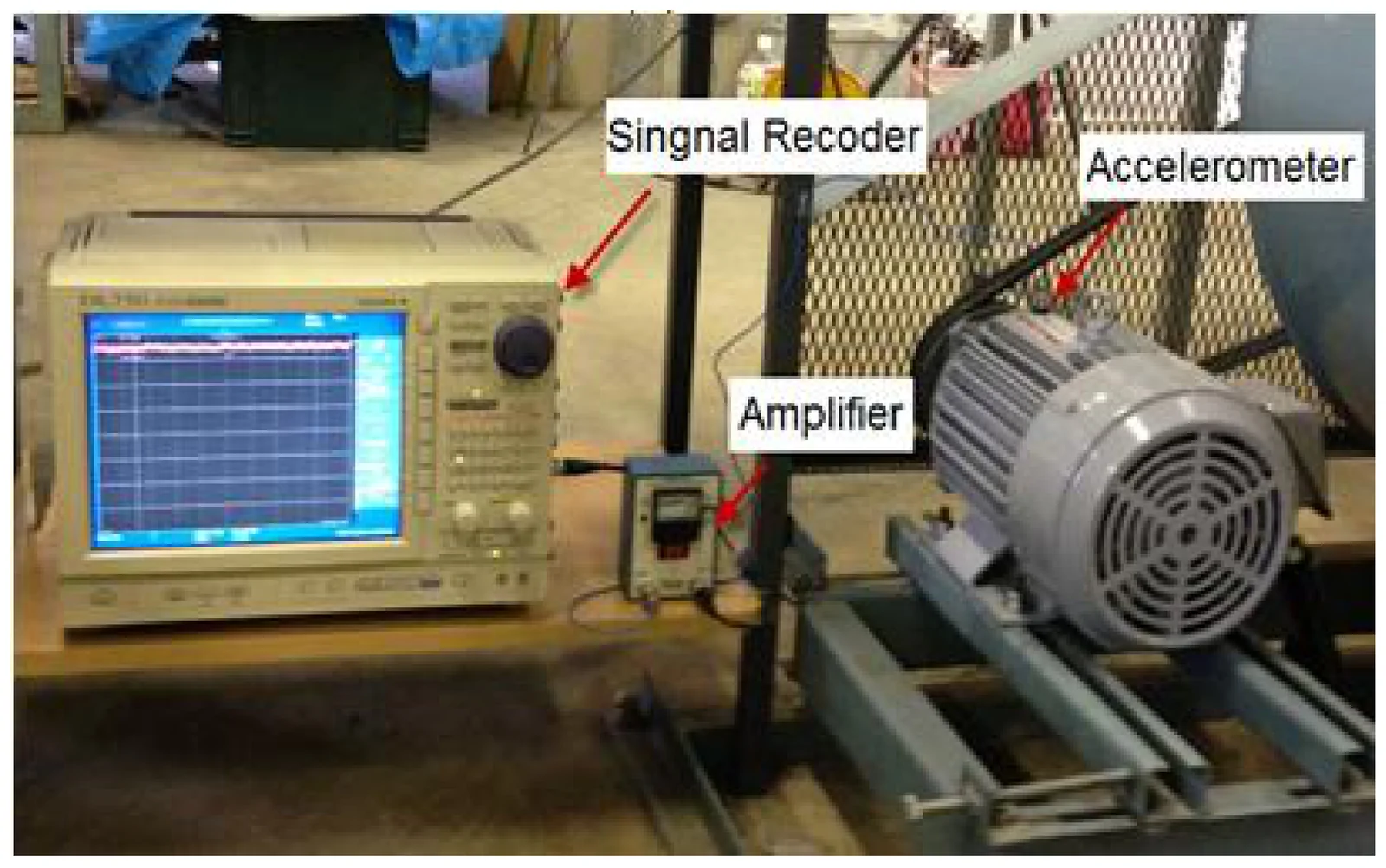
Rolling bearing test platform of JNU [34].

The rolling bearing experimental platform from JNU [34,35] is presented in Figure 6. The platform is designed for fault diagnosis of a centrifugal fan system powered by a Mitsubishi SB-JR induction motor. The motor is a 3.7 kW three-phase induction machine with a rated voltage of 220 V, four poles, and a rated speed of 1800 rpm. The rotor is supported by two bearings, and a faulty bearing is installed as part of the experimental system.

Two types of bearings, N205 and NU205, were adopted in the tests. The N205 bearing was used for the normal condition, outer-race fault, and rolling-element fault, whereas the NU205 bearing, which has a separable outer ring, was selected for the inner-race fault condition. All bearing defects were artificially produced using wire-cutting. For each fault category, vibration data were collected under three rotational speeds, namely 600 rpm, 800 rpm, and 1000 rpm. The sampling frequency was set to 50 kHz, and each data acquisition process lasted 20 s.

#### 3.1.3. Southeast University Dataset

**Figure 7:**
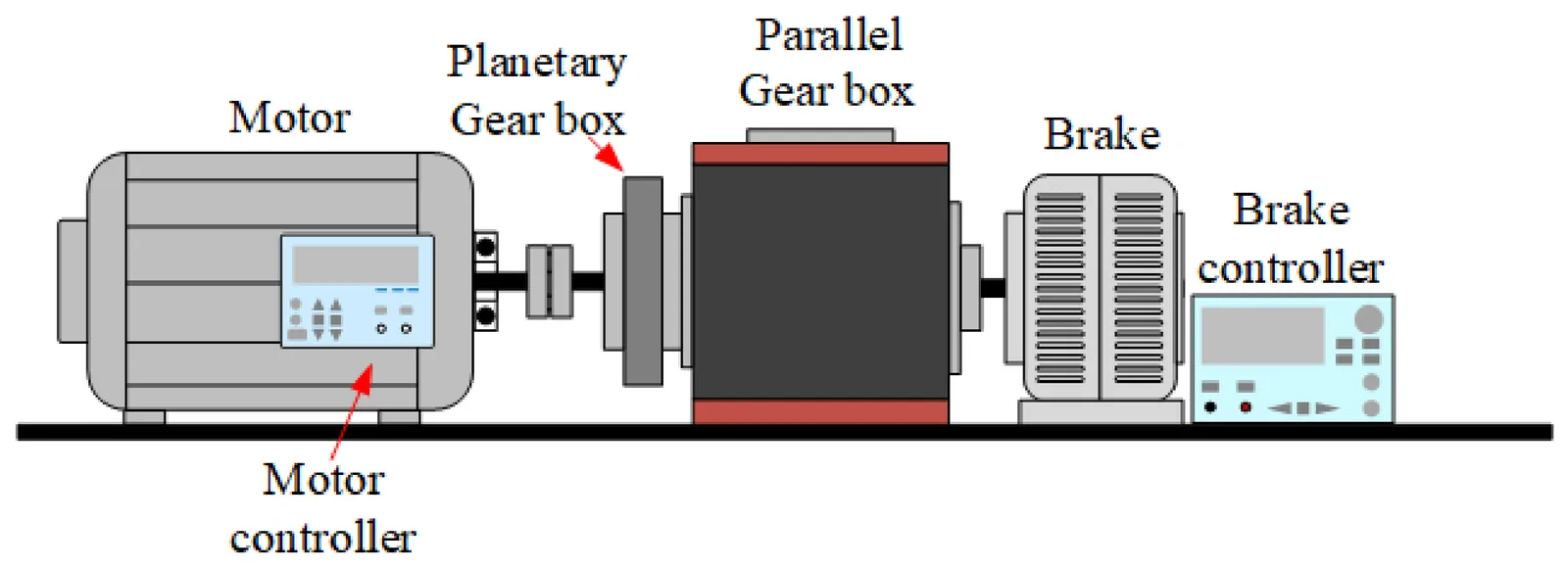
Rolling bearing test platform of SEU [36].

The SEU rolling bearing test platform [36] is shown in Figure 7. The experimental system mainly consists of six parts: a motor controller, an electric motor, a planetary gearbox, a reduction gearbox, a load unit, and a load controller. Seven 608A11 vibration sensors were arranged to acquire vibration signals in the X-, Y-, and Z-directions of both the planetary gearbox and the reduction gearbox, as well as in the Z-direction of the motor. The sampling frequency was set to 5120 Hz.

This dataset contains five bearing health conditions and five gear health conditions under two operating cases. The first case is 20 Hz (1200 rpm) without load, corresponding to 0 V and 0 Nm, while the second case is 30 Hz (1800 rpm) with load, corresponding to 2 V and 7.32 Nm.

### 3.2. Construction of Training and Test Sets

For the CWRU dataset, vibration signals recorded at a shaft speed of 1797 rpm are used. The selected samples cover normal operation and three drive-end bearing fault types, including faults in the inner race, outer race, and rolling element. Each operating condition contains 245,760 signal data points.

For the JNU dataset, signals acquired at 800 rpm are selected. Four operating states are considered, including the normal state and inner-race, outer-race, and rolling-element faults of the drive-end bearing. The signal length corresponding to each state is 500,500 data points.

For the SEU dataset, the data collected at 1200 rpm are adopted. The selected conditions consist of the normal state, inner-race fault, outer-race fault, rolling-element fault, and compound fault. Each condition contains 1,048,560 data points.

To generate samples suitable for deep learning, the continuous vibration signals are segmented using a sliding-window strategy. For each operating condition, a window of 2048 sampling points is moved along the original signal with a stride of 1000 points to generate an initial pool of candidate samples.

To avoid potential information leakage caused by overlapping sliding windows, the training, validation, and test subsets are constructed under a non-overlapping constraint. Specifically, candidate samples are first assigned to the training, validation, and test sets according to the required sample numbers. The overlap of the original signal intervals between different subsets is then examined. Whenever a sample shares any signal points with a sample belonging to another subset, it is discarded and replaced by another candidate sample from the remaining pool until no overlapping signal points exist across the three subsets. Consequently, each fault category contains 60 training samples, 10 validation samples, and 30 test samples for each fault category, thereby guaranteeing complete signal independence among the three subsets. The validation set is used exclusively for BKA-based parameter optimization of the LSSVM classifier, whereas the test set is reserved solely for the final performance evaluation. Samples discarded during the overlap-removal procedure are not used in any stage of model training, parameter optimization, or testing.

**Figure 8:**
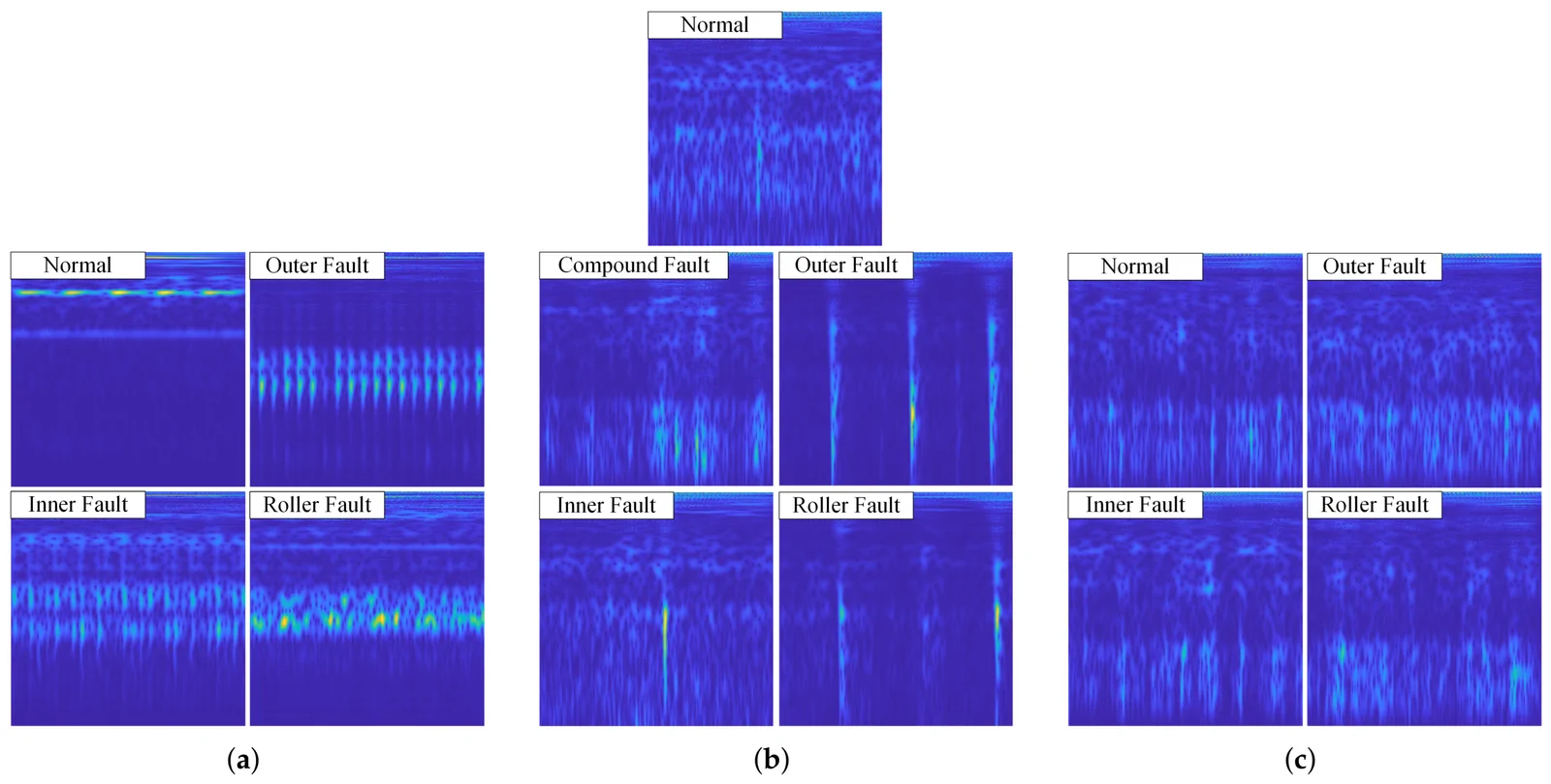
Time–frequency spectrogram of bearing fault signals. (**a**) CWRU dataset. (**b**) SEU dataset. (**c**) JNU dataset.

CWT is subsequently applied to construct the input features. The scale sequence is fixed at 256 for all datasets, while the corresponding physical frequencies are determined according to the sampling frequency of each dataset. Specifically, the sampling frequencies are 12 kHz for the CWRU dataset, 50 kHz for the JNU dataset, and 5.12 kHz for the SEU dataset. For each segmented vibration sample, CWT generates a complex-valued wavelet coefficient matrix, and its magnitude is calculated to obtain the corresponding time–frequency representation, as illustrated in Figure 8. Finally, the resulting time–frequency spectrograms are stored as image files and subsequently used as the inputs for model training, validation, and testing.

In this study, the term “small-sample” is operationally defined as a supervised fault-diagnosis setting in which only a limited number of labeled samples are available for model training. Five progressively reduced training-set sizes are considered, namely 60, 45, 30, 20, and 10 samples per fault class. For all sample-size settings, the validation and test sets are fixed at 10 and 30 samples per class, respectively. The validation set is used exclusively for model selection and BKA-based LSSVM parameter optimization, while the test set is used only for the final performance evaluation.

Specifically, the BKA fitness value is defined as the LSSVM classification accuracy on the fixed validation set. During each optimization iteration, an LSSVM model is trained using the training set, and its fitness is evaluated on the validation set. After the optimal regularization parameter γ and RBF kernel parameter $\sigma^{2}$ are determined, the resulting model is evaluated on the fixed test set. Neither the validation samples nor the test samples are involved in CNN training or final classifier fitting. Samples not selected for the current training-size setting are excluded from that experiment.

Since the CWRU, JNU, and SEU datasets contain 10, 4, and 5 classes, respectively, the total numbers of training samples are 600/450/300/200/100 for CWRU, 240/180/120/80/40 for JNU, and 300/225/150/100/50 for SEU. The corresponding validation-set sizes are 100, 40, and 50, while the test-set sizes are 300, 120, and 150, respectively. Detailed sample settings are presented in Table 1, Table 2 and Table 3.

### 3.3. Model Parameter Settings

On the basis of the established fault diagnosis framework, the main model parameters are defined in this section. The initial CNN architecture and training-related parameters were selected with reference to commonly used configurations reported in related bearing fault diagnosis studies and were subsequently refined through preliminary experiments to achieve stable convergence and avoid overfitting. Once determined, these parameters were kept fixed in all subsequent experiments. The validation and test samples were not used during this preliminary parameter-adjustment process. The architecture-related parameters of the CNN are provided in Table 4. For the LSSVM classifier, its two key parameters, γ and $\sigma^{2}$, are optimized adaptively by BKA. Using the validation-set classification accuracy as the fitness criterion, BKA conducts 50 iterations within the preset search range to identify the parameter combination that leads to the best diagnostic performance. By adopting this optimization strategy, manual parameter tuning is eliminated, while the generalization capability and diagnostic accuracy of the model are improved.

### 3.4. Training and Testing

The number of training epochs for the proposed method was set to 30. Network parameters were updated using the Adam optimizer with an initial learning rate of 0.001. A learning-rate decay scheme with a decay factor of 0.01 was also applied to improve the convergence process. All experiments were implemented on a Windows 10 computer equipped with an AMD R7 7840H processor and an NVIDIA GeForce RTX 4060 GPU.

**Figure 9:**
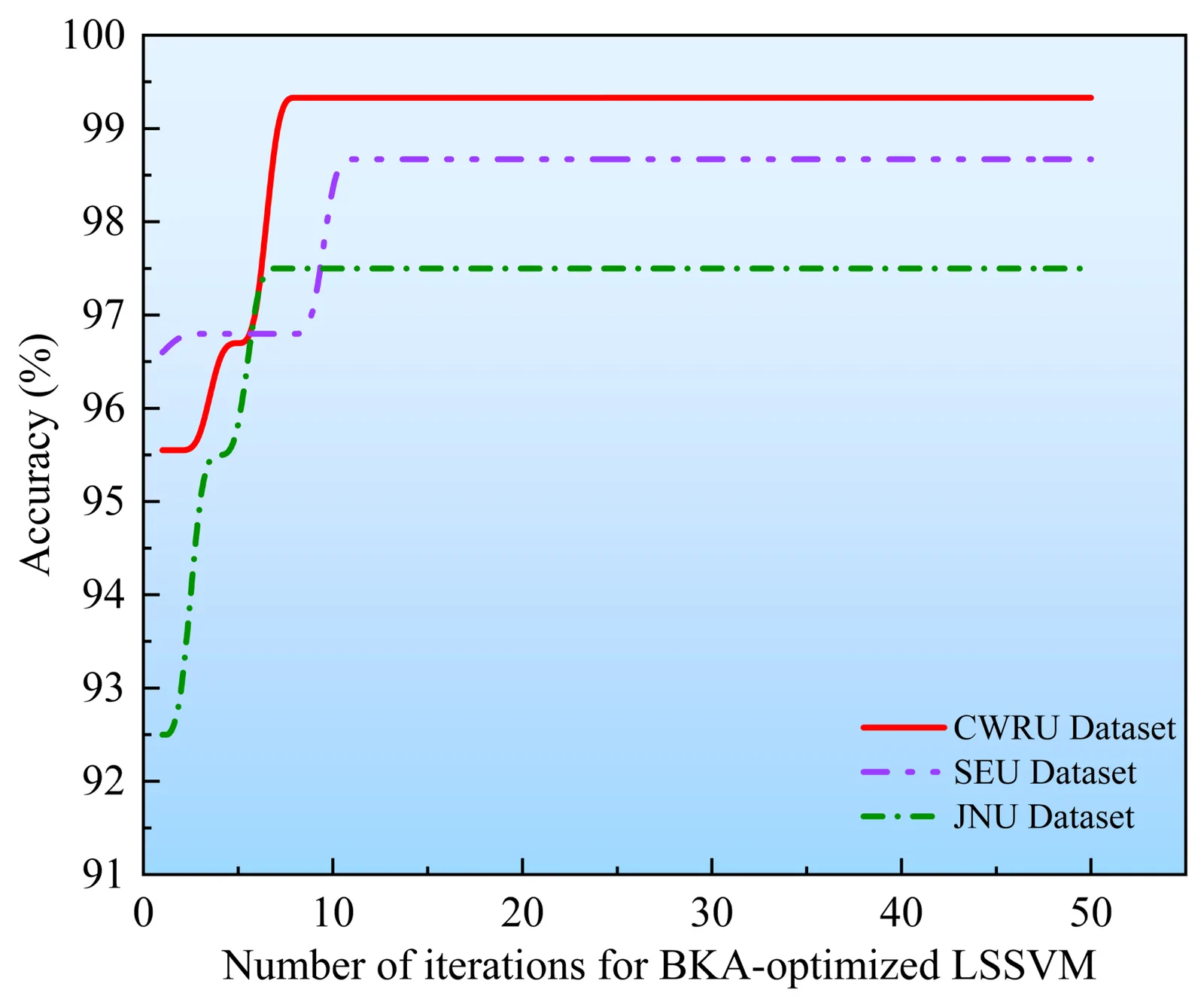
Parameter optimization of LSSVM using the BKA algorithm.

**Figure 10:**
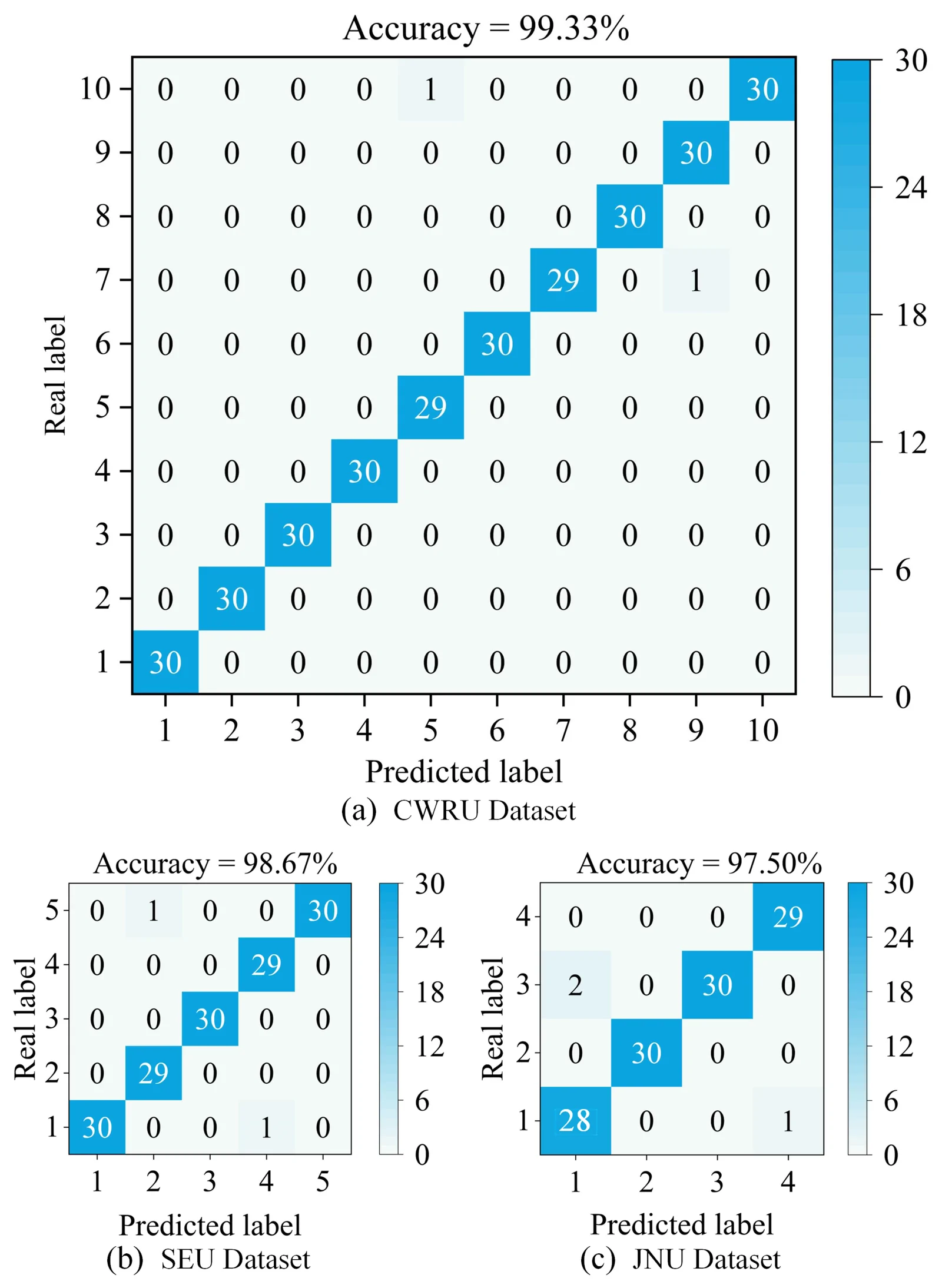
Confusion matrix of classification accuracy.

After CNN training, BKA was applied to determine the optimal LSSVM parameters over 50 optimization iterations, as presented in Figure 9. The optimization curves show that, for all three datasets, the LSSVM classification accuracy exceeded 97% after approximately 10 iterations. This result demonstrates that BKA can efficiently identify suitable parameter combinations. The final diagnostic performance was further assessed using the confusion matrices in Figure 10, where the rows correspond to the predicted labels and the columns indicate the actual labels. The proposed method achieved an accuracy above 97% on each dataset, demonstrating strong recognition performance and adaptability across different bearing fault datasets.

To further examine the robustness of the proposed method, the complete training and testing procedure was independently repeated ten times under the setting of 60 training samples per fault category. In each run, the CNN parameters and optimizer states were reinitialized, the training samples were reshuffled, and the BKA population was independently generated. The remaining training settings were kept unchanged. Macro-F1 was selected as the evaluation metric to reduce the influence of category-dependent performance differences and provide a balanced assessment across fault classes.

**Figure 11:**
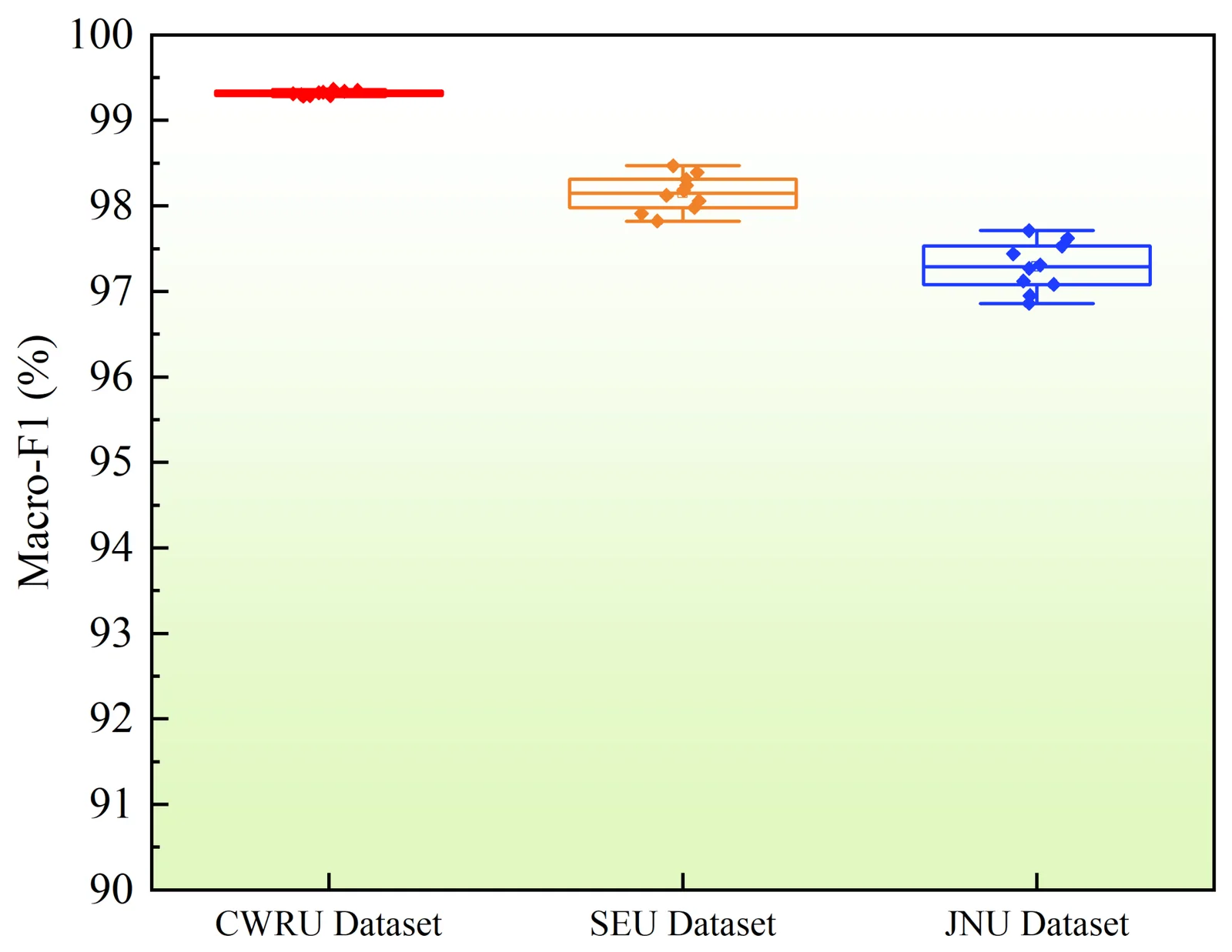
Macro-F1 distributions of the proposed method.

Figure 11 presents the Macro-F1 distributions from ten independent runs on the three datasets. The proposed method shows stable overall diagnostic performance: results on CWRU are highly concentrated, while those on SEU and JNU are broader. This difference is mainly associated with the separability of fault characteristics in each dataset. However, no run shows obvious performance decline, indicating that the method is robust to stochastic training fluctuations and not dependent on a single favourable run.

**Figure 12:**
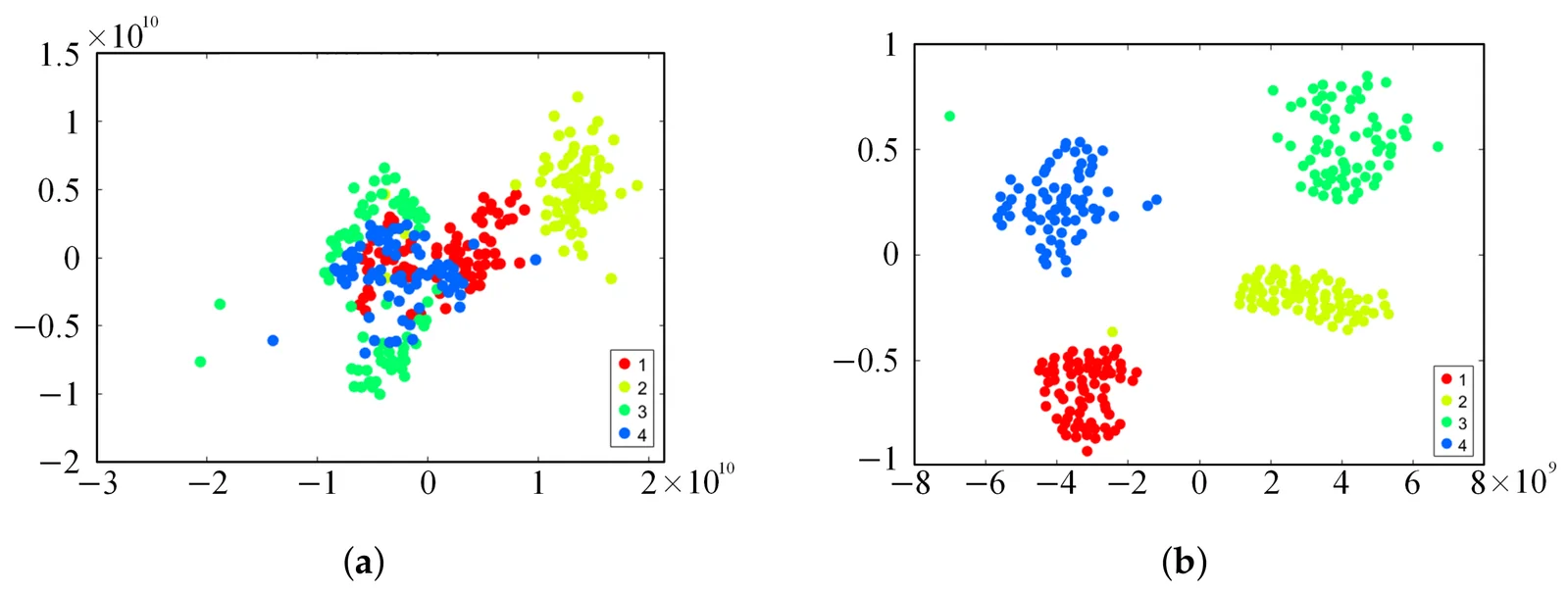
Data distribution. (**a**) Before classification. (**b**) After classification.

To more intuitively evaluate the diagnostic performance of the method, this paper employs t-SNE to perform dimensionality reduction and visualization analysis on both the input features of the test set and the high-level features extracted by the method. Figure 12a displays the distribution of the original test samples after t-SNE dimensionality reduction, reflecting the inherent overlap and separability among various classes before being processed by the method. Figure 12b presents the distribution of features processed by the proposed method in the same low-dimensional space, allowing for a direct observation of whether the method can effectively separate different fault states. The comparison between the two figures clearly illustrates the method’s capability in feature learning and class discrimination.

### 3.5. Ablation Study of the Proposed Framework

To further clarify the individual contribution of each component in the proposed framework, an ablation study was conducted on the CWRU, JNU, and SEU datasets under the small-sample setting. Specifically, 60 samples per class were used for training, while the validation and test sets were fixed at 10 and 30 samples per class, respectively. Five representative models were constructed by progressively introducing different components of the proposed framework, namely Raw-1D-CNN-Softmax, CWT-PCA-LSSVM (Grid), CWT-CNN-Softmax, CWT-CNN-LSSVM (Grid) and CWT-CNN-BKA-LSSVM. For the CWT-PCA-LSSVM (Grid) model, PCA was employed for feature extraction, and the retained principal components preserved 95% of the cumulative explained variance. Apart from the investigated component, all remaining experimental settings, including the CNN architecture, training strategy, LSSVM configuration, and data partitions, were kept identical to ensure a fair comparison. Each model was independently evaluated over ten repeated runs.

**Figure 13:**
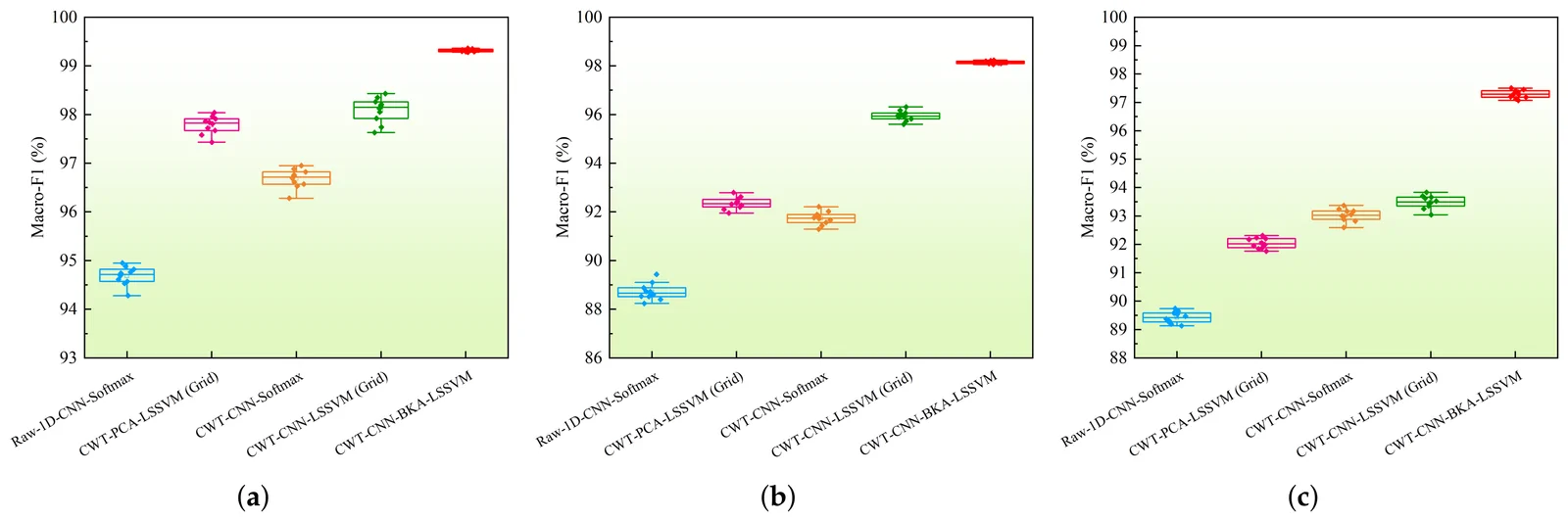
Ablation study of the proposed method. (**a**) CWRU dataset. (**b**) SEU dataset. (**c**) JNU dataset.

As shown in Figure 13, the proposed CWT-CNN-BKA-LSSVM consistently achieves the highest median Macro-F1 and exhibits one of the smallest performance variations on all three datasets. Compared with the Raw-1D-CNN-Softmax model, introducing CWT time–frequency preprocessing substantially improves the diagnostic performance. This improvement indicates that the time–frequency representation generated by CWT preserves more discriminative fault characteristics than raw vibration signals, thereby providing a more informative input for subsequent feature learning.

The contribution of CNN-based feature learning can be observed by comparing the CWT-PCA-LSSVM (Grid) and CWT-CNN-LSSVM (Grid) models. Although both methods employ the same CWT preprocessing and LSSVM classifier, replacing handcrafted PCA features with automatically learned CNN features leads to a clear improvement in Macro-F1 on all three datasets. This result demonstrates that the proposed CNN architecture is capable of extracting more discriminative and robust fault representations from the CWT time–frequency images, particularly under limited training samples.

The effectiveness of the LSSVM classifier is verified by comparing CWT-CNN-Softmax with CWT-CNN-LSSVM (Grid). Replacing the conventional Softmax classifier with LSSVM consistently improves the classification performance, indicating that the margin-based decision mechanism of LSSVM provides better generalization ability than Softmax when only a limited number of labeled samples are available.

The contribution of the proposed BKA optimization strategy is demonstrated by comparing CWT-CNN-BKA-LSSVM with CWT-CNN-LSSVM (Grid). The BKA optimization method improves the LSSVM classifier to some extent and achieves the highest Macro-F1 across the three datasets. Moreover, its boxplots exhibit relatively smaller interquartile ranges and shorter whiskers, indicating lower run-to-run variability and more stable parameter optimization.

Overall, the ablation results demonstrate that each component contributes positively to the final diagnostic performance. Specifically, CWT enhances the representation of non-stationary vibration signals, CNN extracts more discriminative deep features, LSSVM improves the classification capability under limited training samples, and BKA further enhances both optimization accuracy and robustness. The combination of these four components enables the proposed CWT-CNN-BKA-LSSVM framework to achieve the best overall performance on all three bearing datasets.

### 3.6. Comparison of Different Optimization Algorithms

To further validate the performance advantage of the BKA in the LSSVM parameter optimization task, this study conducts a comparative experiment by contrasting BKA with two classic meta-heuristic optimization algorithms—PSO and GA—while keeping the rest of the model structure and training settings unchanged. In the experiment, the three algorithms are respectively employed to optimize the regularization parameter γ and the kernel parameter $\sigma^{2}$ of LSSVM, and fault diagnosis tests are performed on three public bearing datasets.

To ensure the fairness of the comparison, all optimization algorithms are configured using identical optimization budgets. BKA, PSO, and GA employ the same population size (20), maximum iteration number (50), parameter search ranges ($\gamma ,\sigma^{2}\in \left[10^{-6},10^{6}\right]$), random initialization strategy, and validation-based fitness function. Consequently, the differences in diagnostic performance are attributed to the optimization mechanisms of the algorithms rather than unequal computational resources.

**Figure 14:**
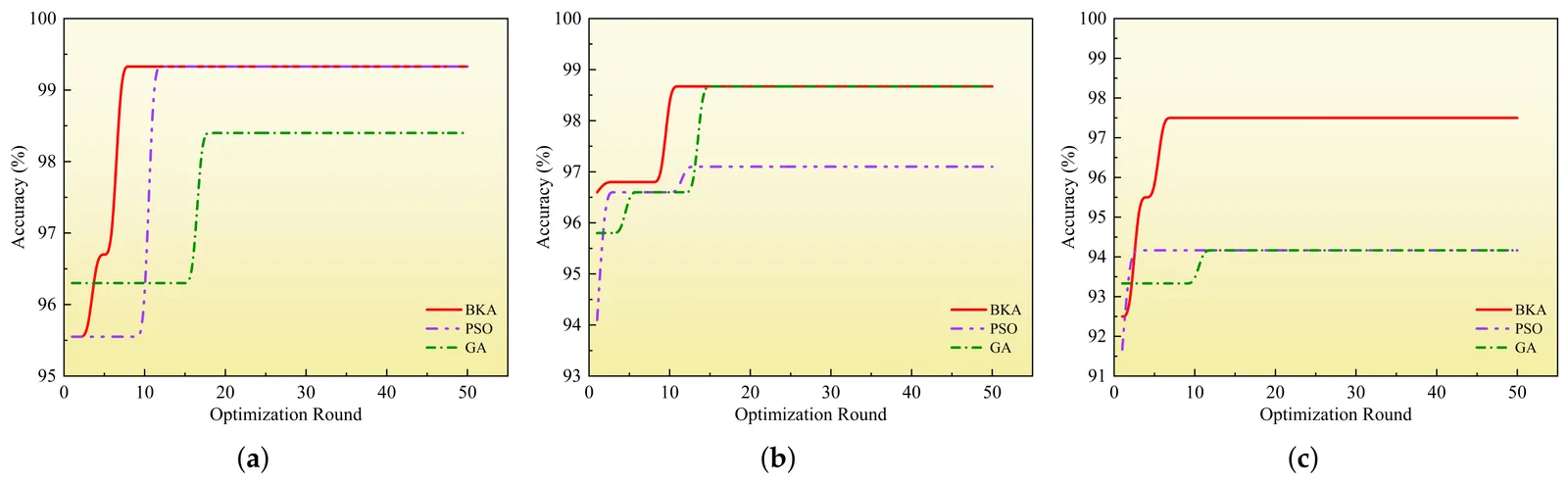
Comparison of different optimization algorithms. (**a**) CWRU dataset. (**b**) SEU dataset. (**c**) JNU dataset.

As shown in Figure 14, the experimental results indicate that

(1) In terms of convergence speed, BKA tends to stabilize after approximately 10 iterations, whereas PSO and GA usually require more iterations to reach a similar level.
(2) In terms of final optimization performance, the LSSVM classifier optimized by BKA achieves the highest stable accuracy on all three datasets.

Overall, BKA outperforms PSO and GA in both optimization efficiency and final classification accuracy. This verifies its fast convergence and strong global optimization capability in solving the LSSVM parameter optimization problem and highlights its unique advantages in this application scenario.

### 3.7. Small-Sample Comparative Experiment

To formally evaluate the diagnostic performance under limited data, this paper considers five progressively reduced training-set sizes, with the specific dataset partitions presented in Table 1, Table 2 and Table 3. To verify the generalization performance of the proposed method in industrial scenarios with limited samples, a comparative experiment under small-sample conditions is designed in this study, with CNN, CNN-SVM, and CNN-BiGRU selected as benchmark comparisons and the parameter settings for each model shown in Table 5, Table 6 and Table 7. The hyperparameter settings of the baseline models are determined by referring to the recommended configurations reported in the corresponding literature and are further manually adjusted through preliminary experiments to achieve stable performance on the considered datasets. Once determined, these hyperparameters are kept unchanged for all experiments to ensure a fair comparison.

All comparison methods used the same 128×128 pixel time–frequency images generated through the identical CWT preprocessing procedure. For each training-set size, the complete training, validation, parameter-optimization, and testing procedure was independently repeated ten times. In each run, the training samples were selected using stratified random sampling, and the model parameters, mini-batch order, and stochastic optimization components were independently initialized. The validation and test sets remained unchanged across all runs. To ensure a fair comparison, all methods used the same training subsets, validation set, test set, and random seeds within each run. The results are reported as the mean Macro-F1 score, with the error bars representing one standard deviation over the ten independent runs.

**Figure 15:**
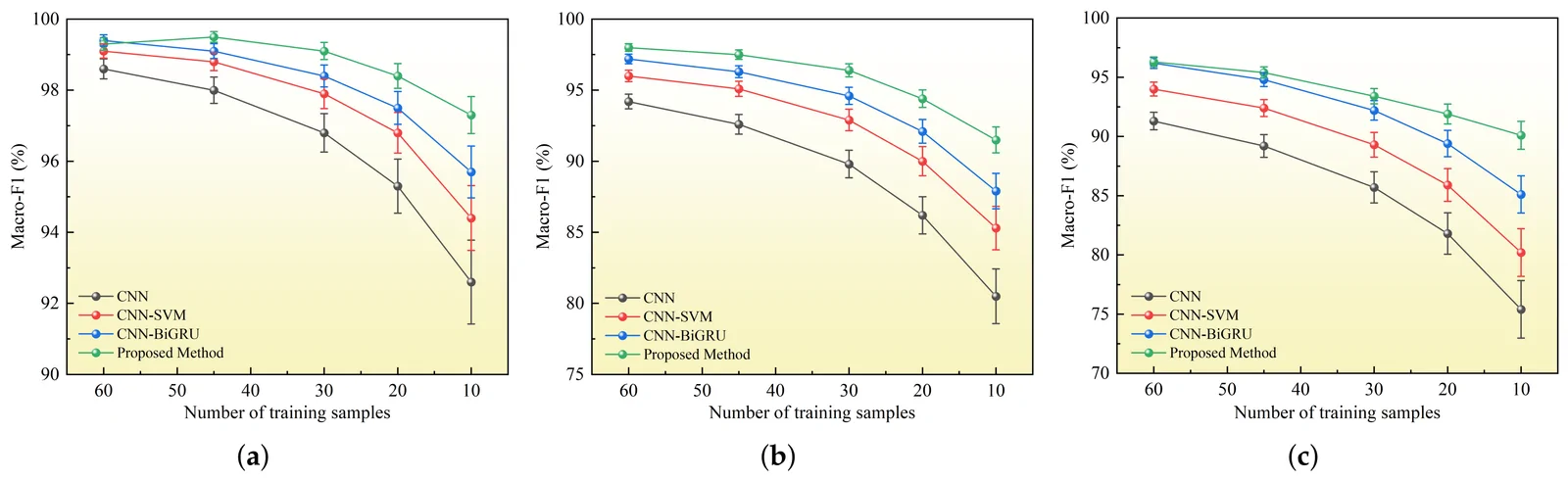
Repeated-run Macro-F1 performance of different diagnostic methods. (**a**) CWRU dataset. (**b**) SEU dataset. (**c**) JNU dataset.

Figure 15 compares repeated-run Macro-F1 results of CNN, CNN-SVM, CNN-BiGRU, and the proposed CNN-BKA-LSSVM on three datasets under progressively reduced training sizes. Across all datasets, Macro-F1 decreases with fewer samples, confirming that limited labels hinder discriminative learning. On CWRU, all methods perform well with ≥30 samples/class, but differences grow at 20 and 10 samples; the proposed method shows the smallest degradation, highest F1, and tighter error bars, indicating less sensitivity to random selection and initialization. Trends on JNU and SEU are similar but more pronounced: CNN and CNN-SVM degrade substantially, CNN-BiGRU is more robust yet inferior to the proposed method, and the proposed method retains much higher F1 with smaller error bars under 10 samples. Dataset discrepancies arise from fault separability. As indicated in Figure 8, the fault categories in the CWRU dataset exhibit the clearest distinctions, while those in the SEU dataset remain moderately separable. In contrast, the time–frequency patterns of the JNU dataset are less distinct and show greater overlap, making fault classification more difficult. Nevertheless, the proposed method consistently achieves the best average F1. Overall, it not only performs well under standard settings but also degrades more slowly and with lower variability as sample size reduces, providing evidence for its effectiveness and robustness in small-sample bearing fault diagnosis.

To further investigate the feature-learning capability of the four methods under the small-sample setting, t-SNE was employed to visualize the learned feature distributions. In this experiment, 20 samples per class were used for training, 10 samples per class were reserved for validation during BKA optimization, and the remaining 70 samples per class were projected into the two-dimensional t-SNE space for visualization. Since the purpose of this experiment is qualitative feature analysis rather than quantitative performance evaluation, a larger visualization set was intentionally adopted to obtain denser feature distributions and more reliable cluster structures.

**Figure 16:**
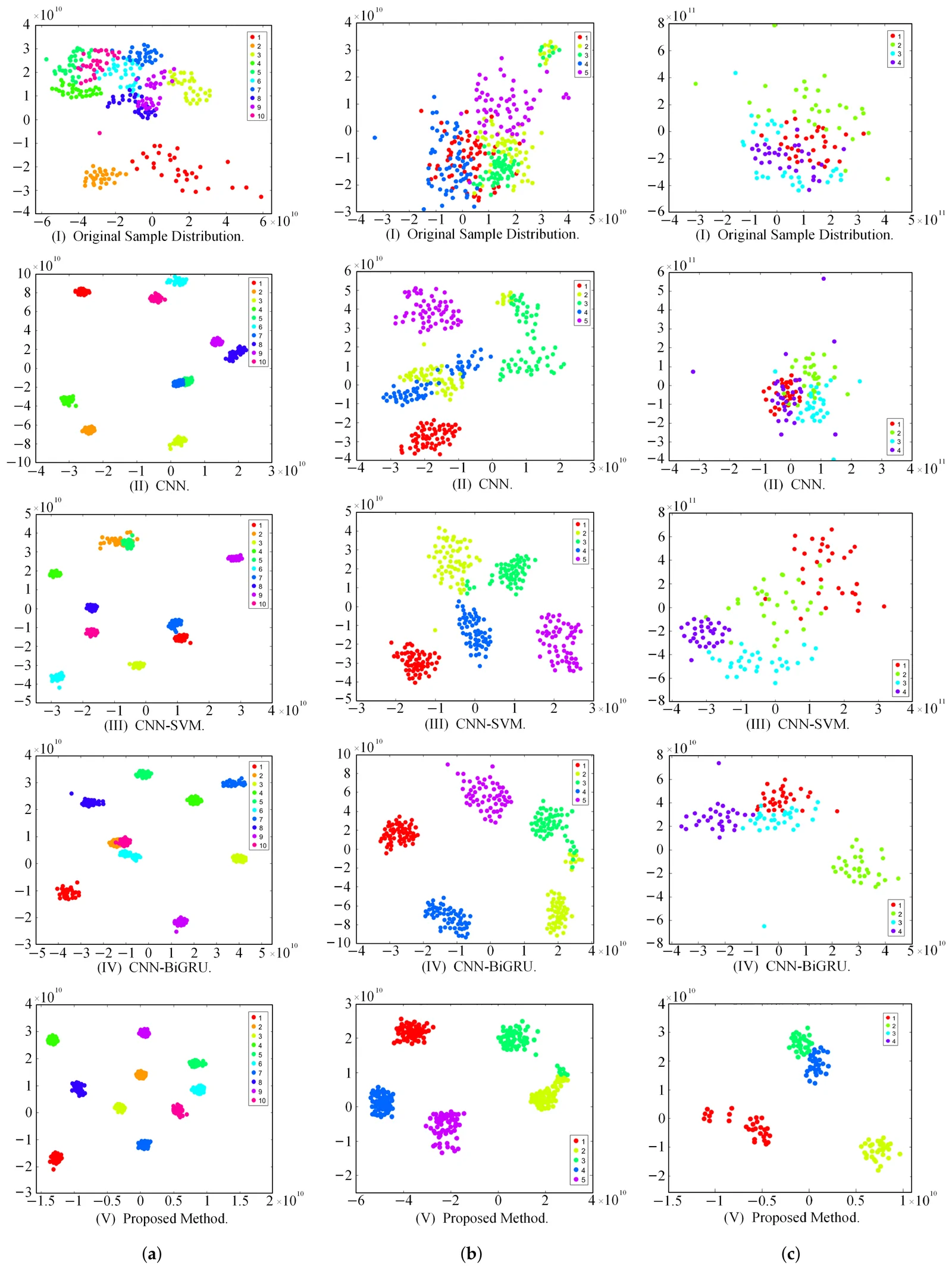
Feature distributions extracted by each method. (**a**) CWRU dataset. (**b**) SEU dataset. (**c**) JNU dataset.

As shown in Figure 16, the original data present varying levels of overlap among fault classes, indicating that raw vibration samples alone cannot adequately distinguish different operating states. After feature learning, class separability is improved by all methods to different degrees. Nevertheless, the proposed approach consistently produces more compact intra-class distributions and wider inter-class distances on all three datasets. For the CWRU and SEU datasets, it further clarifies the class boundaries and generates more regular feature distributions. Its advantage is particularly evident on the JNU dataset, where the extracted features form the clearest clusters, with reduced class overlap, tighter samples within each class, and larger margins between different classes. These observations demonstrate that CWT-CNN-BKA-LSSVM can learn highly discriminative fault representations from limited samples and achieves better within-condition diagnostic performance and stability on independent test samples, particularly for datasets with complex and overlapping fault features.

## 4. Conclusions

In this study, a CWT-CNN-BKA-LSSVM method for small-sample bearing fault diagnosis was constructed and validated. In this method, CNN is used to extract deep fault features, while the LSSVM classifier optimized by BKA is employed to identify fault states. Experimental validation on three public datasets and comparisons with several typical diagnostic methods led to the following conclusions:

(1) The proposed CWT-CNN-BKA-LSSVM achieved better diagnostic performance than the comparative methods under small-sample conditions. The results indicate that, compared with CNN, CNN-SVM, and other related models, the BKA-optimized LSSVM classifier can more effectively handle the high-dimensional features learned by CNN, showing improved generalization and classification robustness when training samples are limited.
(2) The use of BKA for adaptive LSSVM parameter optimization further enhanced the overall model performance. The comparison with other optimization algorithms demonstrates that BKA converges faster and provides stronger global search ability than PSO and GA when optimizing the regularization parameter γ and kernel parameter $\sigma^{2}$ of LSSVM. This helps overcome the inefficiency and local optimum problems commonly associated with manual parameter selection.
(3) The experiments on different datasets, together with visualization analysis, confirm the effectiveness of the method under the evaluated operating conditions of the proposed method. The t-SNE results show that, after CNN feature extraction and BKA-LSSVM-optimized classification, different fault classes present more compact intra-class distributions and clearer inter-class separation in the low-dimensional space. This advantage is particularly evident on datasets with less distinguishable fault features, such as the JNU dataset, where the proposed method produces more distinct clusters and clearer class boundaries. Overall, the classification results and feature distributions demonstrate that the proposed method performs well in diagnostic accuracy, small-sample adaptability, and feature separability, providing a useful reference for bearing fault diagnosis in industrial scenarios with limited fault data.

Despite the encouraging results, further validation is still needed. The experiments were conducted on public benchmark datasets under controlled conditions, and the performance of the proposed method under more complex industrial environments, such as variable operating conditions, strong noise, and cross-machine scenarios, remains to be examined. In addition, the CWT, CNN, and BKA procedures introduce a certain computational cost, while imbalanced, unknown, and compound fault conditions were not fully considered in this study.

Future work will focus on industrial and cross-domain validation, while exploring transfer learning, data augmentation, and lightweight model designs to improve robustness and computational efficiency. Unknown-fault recognition, multi-sensor fusion, and online diagnosis will also be further investigated.

## Figures and Tables

**Table 1:** CWRU dataset.

Fault Type	Training	Validation	Test	Label
Normal	60/45/30/20/10	10	30	1
0.007-inch Inner Race	60/45/30/20/10	10	30	2
0.007-inch Roller	60/45/30/20/10	10	30	3
0.007-inch Outer Race	60/45/30/20/10	10	30	4
0.014-inch Inner Race	60/45/30/20/10	10	30	5
0.014-inch Roller	60/45/30/20/10	10	30	6
0.014-inch Outer Race	60/45/30/20/10	10	30	7
0.021-inch Inner Race	60/45/30/20/10	10	30	8
0.021-inch Roller	60/45/30/20/10	10	30	9
0.021-inch Outer Race	60/45/30/20/10	10	30	10

**Table 2:** JNU dataset.

Fault Type	Training	Validation	Test	Label
Normal	60/45/30/20/10	10	30	1
NU205 Inner Race	60/45/30/20/10	10	30	2
N205 Roller	60/45/30/20/10	10	30	3
N205 Outer Race	60/45/30/20/10	10	30	4

**Table 3:** SEU dataset.

Fault Type	Training	Validation	Test	Label
Normal	60/45/30/20/10	10	30	1
Inner Race	60/45/30/20/10	10	30	2
Roller	60/45/30/20/10	10	30	3
Outer Race	60/45/30/20/10	10	30	4
Compound Fault	60/45/30/20/10	10	30	5

**Table 4:** CNN parameter configuration of the proposed method.

Layer Type	Parameter	Value
Conv1	Kernel/Filters	3 × 3/16
Batchnorm1	Quantity	16
Activation	Relu	-
Pool1	Pool Size/Stride	2 × 2/2
Dropout1	Dropout Rate	0.2
Conv2	Kernel/Filters	5 × 5/32
Batchnorm2	Quantity	32
Activation	Relu	-
Pool2	Pool Size/Stride	2 × 1/2
Dropout2	Dropout Rate	0.1
Fullconnect1	Quantity	64
Dropout3	Dropout Rate	0.1
Fullconnect2	Quantity	32
Dropout4	Dropout Rate	0.1
Fullconnect3	Output Neurons	10 (CWRU)/5 (SEU) /4 (JNU)

**Table 5:** CNN parameter settings.

Parameter	Value
Convolutional Modules	Identical to Proposed Method’s CNN Layer
Classifier	Softmax

**Table 6:** CNN-SVM parameter settings.

Parameter	Value
Convolutional Modules	Identical to Proposed Method’s CNN Layer
Kernel	RBF
Penalty parameter *C*	0.01
Nuclear parameter γ	3000

**Table 7:** CNN-BiGRU parameter settings.

Parameter	Value
Convolutional Modules	Identical to Proposed Method’s CNN Layer
Number of GRU layers	2
Number of hidden units	25
Sequence processing direction	Two-way
Sequence input form	Flattened feature vector
Sequence length	Self-adaptive
Two-way integration method	Concatenation
Output dimension	50

## Data Availability

The raw data analyzed in this study were obtained from publicly available bearing fault datasets, including the Case Western Reserve University (CWRU) Bearing Data Center, the Jiangnan University (JNU) bearing dataset, and the Southeast University (SEU) mechanical dataset. The CWRU dataset is available at https://engineering.case.edu/bearingdatacenter (accessed on 14 March 2025); the JNU bearing dataset is available at https://github.com/ClarkGableWang/JNU-Bearing-Dataset (accessed on 25 October 2025); and the SEU mechanical dataset is available at https://github.com/Yxz3930/SEU-datasets (accessed on 25 October 2025). The processed time–frequency images and experimental results generated during this study are available from the first author or the corresponding author upon reasonable request.
